# Autoimmune encephalitis: proposed recommendations for symptomatic and long-term management

**DOI:** 10.1136/jnnp-2020-325302

**Published:** 2021-03-01

**Authors:** Hesham Abboud, John Probasco, Sarosh R Irani, Beau Ances, David R Benavides, Michael Bradshaw, Paulo Pereira Christo, Russell C Dale, Mireya Fernandez-Fournier, Eoin P Flanagan, Avi Gadoth, Pravin George, Elena Grebenciucova, Adham Jammoul, Soon-Tae Lee, Yuebing Li, Marcelo Matiello, Anne Marie Morse, Alexander Rae-Grant, Galeno Rojas, Ian Rossman, Sarah Schmitt, Arun Venkatesan, Steven Vernino, Sean J Pittock, Maarten Titulaer, Autoimmune Encephalitis Alliance Clinicians Network, Rawan Tarawneh

**Affiliations:** 1 Neurology, Case Western Reserve University, Cleveland, Ohio, USA; 2 Multiple Sclerosis and Neuroimmunology Program, University Hospitals of Cleveland, Cleveland, Ohio, USA; 3 Neurology, Johns Hopkins Medicine, Baltimore, Maryland, USA; 4 Oxford Autoimmune Neurology Group, John Radcliffe Hospital, Oxford, UK; 5 Neurology, Washington University in St Louis, St Louis, Missouri, USA; 6 Neurology, University of Maryland School of Medicine, Baltimore, Maryland, USA; 7 Neurology, Rosalind Franklin University of Medicine and Science, North Chicago, Illinois, USA; 8 Neurology, Billings Clinic, Billings, Montana, USA; 9 Neurology, Minas Gerais Federal University Risoleta Tolentino Neves Hospital, Belo Horizonte, Brazil; 10 Neuroimmunology Group, The University of Sydney Faculty of Medicine and Health, Sydney, New South Wales, Australia; 11 Neurology, La Paz University Hospital, Madrid, Spain; 12 Neurology, Mayo Clinic, Rochester, Minnesota, USA; 13 Neurology, Tel Aviv Sourasky Medical Center, Tel Aviv, Israel; 14 Neurology, Cleveland Clinic, Cleveland, Ohio, USA; 15 Neurology, Northwestern University Feinberg School of Medicine, Chicago, Illinois, USA; 16 Neurology, Seoul National University College of Medicine, Seoul, The Republic of Korea; 17 Neurology, Massachusetts General Hospital, Boston, Massachusetts, USA; 18 Neurology, Harvard Medical School, Boston, Massachusetts, USA; 19 Pediatric Neurology, Geisinger Commonwealth School of Medicine, Scranton, Pennsylvania, USA; 20 Neurology, Sanatorio de La Trinidad Mitre, Buenos Aires, Argentina; 21 Favaloro Foundation, Buenos Aires, Argentina; 22 Neuro-developmental Science Center, Akron Children's Hospital, Akron, Ohio, USA; 23 Neurology, MUSC, Charleston, South Carolina, USA; 24 Neurology, UT Southwestern, Dallas, Texas, USA; 25 Neurology, Erasmus Medical Center, Rotterdam, Zuid-Holland, Netherlands

**Keywords:** autoimmune encephalitis, neuroimmunology, paraneoplastic syndrome

## Abstract

The objective of this paper is to evaluate available evidence for each step in autoimmune encephalitis management and provide expert opinion when evidence is lacking. The paper approaches autoimmune encephalitis as a broad category rather than focusing on individual antibody syndromes. Core authors from the Autoimmune Encephalitis Alliance Clinicians Network reviewed literature and developed the first draft. Where evidence was lacking or controversial, an electronic survey was distributed to all members to solicit individual responses. Sixty-eight members from 17 countries answered the survey. The most popular bridging therapy was oral prednisone taper chosen by 38% of responders while rituximab was the most popular maintenance therapy chosen by 46%. Most responders considered maintenance immunosuppression after a second relapse in patients with neuronal surface antibodies (70%) or seronegative autoimmune encephalitis (61%) as opposed to those with onconeuronal antibodies (29%). Most responders opted to cancer screening for 4 years in patients with neuronal surface antibodies (49%) or limbic encephalitis (46%) as opposed to non-limbic seronegative autoimmune encephalitis (36%). Detailed survey results are presented in the manuscript and a summary of the diagnostic and therapeutic recommendations is presented at the conclusion.

## Introduction

In the first part of the Proposed Best Practice Recommendations, we covered diagnosis and acute immunotherapy for autoimmune encephalitis (AE). In this second part, we will cover symptomatic, bridging and maintenance immunotherapy of AE. The recommendations are based on literature review and an online survey of 68 members of the Autoimmune Encephalitis Alliance Clinicians Network (AEACN). The final manuscript was approved by all participating members after four rounds of revisions. Please refer to part-1 (Proposed Best Practice Recommendations for Diagnosis and Acute Management) for methodology details.

### Symptomatic therapy

AE is often polysymptomatic. Symptoms start in the acute phase and may resolve or improve with acute immunotherapy alone or combined with targeted symptomatic treatment. However, many residual symptoms persist beyond the acute phase requiring long-term symptomatic therapy. In this section, we will review symptomatic therapy in both the acute phase of the disease and the long-term. A summary of symptomatic therapy recommendations is included in table 1.

**Table 1:** Symptomatic management for autoimmune encephalitis

Symptom category	Therapeutic options	Precautions
Psychosis/agitation/mania	Acute immunotherapy with IVMP, IVIg and/or PLEX. Benzodiazepines (eg, clonazepam, diazepam). Antipsychotics (eg, quetiapine). Mood stabilisers (eg, valproic acid). Establish safety measures as necessary (eg, bed padding, soft restraints, room sitter).	Avoid over-sedation and unnecessary polypharmacy. Avoid medications that lower seizure threshold in patients with high seizure risk (eg, clozapine, olanzapine). Avoid medications that prolong QT interval in dysautonomic patients (eg, ziprasidone, haloperidol). Watch out for worsening of involuntary movements or development of neuroleptic malignant syndrome.
Seizures	Acute immunotherapy with IVMP, IVIg and/or PLEX. Antiseizure medications (sodium channel blockers like carbamazepine or lacosamide may be preferred in LGI1-antibody encephalitis). Medically induced coma with midazolam, pentobarbital or propofol is required for NORSE.	Instate early immunotherapy for patients with seizures in the setting of suspected AE. Avoid use of anti-seizure medications alone. May cautiously attempt weaning antiseizure medications in patients with early seizure freedom and normal brain MRI and EEG.
Movement disorders	Acute immunotherapy with IVMP, IVIg and/or PLEX. Benzodiazepines (eg, clonazepam, diazepam) for myoclonus, SPS, PERM, catatonia, dystonia, stereotypies and hyperkinesia. Anticholinergics (eg, trihexyphenidyl, benzatropine) for dystonia. Muscle relaxants (eg, baclofen, tizanidine) for dystonia and spasticity. Dopamine blockers (eg, risperidone) or depleters (tetrabenazine) for chorea, athetosis, balism, tics and hyperkinesia. Dopamine agonists (eg, pramipexole, ropinirole) or carbidopa/levodopa for acquired parkinsonism, rigidity and akinetic mutism.	Avoid over-sedation and unnecessary polypharmacy. Watch for paradoxical worsening of involuntary movements or development of neuroleptic malignant syndrome. Practice caution with anticholinergics in patients with dysautonomia. Practice caution with anticholinergics and dopaminergic medications in patients with psychosis.
Dysautonomia	Acute immunotherapy with IVMP, IVIg and/or PLEX. ICU monitoring for severe dysautonomia. Beta-blockers (eg, propranolol), alpha-2 blockers (eg, clonidine), and/or acetylcholine esterase inhibitors (pyridostigmine) for increased sympathetic drive. Midodrine, fludrocortisone or droxidopa for symptomatic postural hypotension. Temporary pacing for heart block or severe arrhythmia. Total parental nutrition for patients with severe gastrointestinal dysmotility. Anti-muscarinics (eg, oxybutynin) for bladder incontinence.	Watch for exaggerated response to sympatholytic therapies. Watch for supine hypertension when treating postural hypotension. Watch for cognitive and cardiac side effects when using antimuscarinics.
Sleep disorders	Acute immunotherapy with IVMP, IVIg and/or PLEX. Promote sleep hygiene and uninterrupted night-time sleep. Melatonin to promote the sleep-wake cycle. Sedating benzodiazepines (eg, temazepam), benzodiazepine receptor agonists (eg, zolpidem) and/or non-benzodiazepine hypnotics (eg, zopiclone) for insomnia. Wake-promoting agents (eg, modafinil) and/or traditional stimulants (eg, methylphenidate) for excessive daytime sleepiness. Evaluate residual sleep disorders with polysomnography and treat sleep disordered breathing if present.	Avoid over-sedation and unnecessary polypharmacy. Practice caution when using stimulants in patients with seizures or hyperkinetic involuntary movements.

AE, autoimmune encephalitis; EEG, electroencephalogram; ICU, intensive care unit; IVIg, intravenous immunoglobulins; IVMP, intravenous methyl-prednisolone; LGI1, leucine-rich glioma inactivated-1; NORSE, new onset refractory status-epilepticus; PERM, progressive encephalomyelitis with rigidity and myoclonus; PLEX, plasma exchange; SPS, stiff person syndrome.

#### Management of psychosis

Often benzodiazepines are required in large doses for adequate sedation. Many patients with AE will need antipsychotics to control agitation and psychosis.^1^ One option is to avoid agents that lower seizure threshold (eg, clozapine and olanzapine)^2^ in patients with seizures or who are at increased seizure risk (eg, patients with limbic or cortical encephalitis or who have lateralised periodic discharges (LPDs) on electroencephalogram (EEG)). Antipsychotics that prolong the QT interval (eg, ziprasidone and IV haloperidol) should be used with caution or avoided in dysautonomic patients with symptomatic bradycardia or heart block. If an antipsychotic results in worsening of agitation or involuntary movements after initiation, it should be stopped and substituted with an alternative agent. In NMDAR-antibody encephalitis (see online supplemental appendix 1 for full names of neuronal autoantibodies (NAAs)), patients may be particularly sensitive to the extrapyramidal side effects of antipsychotics and may experience worsening of catatonia and other involuntary movement or even develop neuroleptic malignant syndrome.^3^ Second generation antipsychotics with the least potential for inducing seizures and extrapyramidal side effects (eg, quetiapine) may be preferred in patients with AE. Patients with manic symptoms in the setting of AE may be treated with mood stabilisers such as valproic acid especially in case of comorbid seizures.^1^ Elimination or dose reduction of certain medications may also improve behavioural symptoms in some patients (eg, steroids, benzodiazepines). It is important to instate safety measures (eg, padding, soft restraints, etc) for agitated patients to prevent self-injury and harm to others.

10.1136/jnnp-2020-325302.supp1 Supplementary data

#### Management of seizures

In addition to immunotherapy, patients with AE with clinical or electrophysiological seizures may require treatment with antiseizure medications effective against focal seizures.^4^ However, in leucine-rich glioma inactivated-1 (LGI1)-antibody encephalitis, despite there being data showing sodium-channel blockers may be the most effective antiseizure medications,^5 6^ it is very clear that immunotherapy is far more effective than seizure medications in general. Hence, immunotherapies should be the antiseizure medication of choice in this condition.^6–9^ Patients with status-epilepticus may require standard status-epilepticus protocol with fast-acting intravenous benzodiazepines followed by intravenous loading of an appropriate antiseizure medication such as fosphenytoin, valproic acid or levetiracetam. Patients with new onset refractory status-epilepticus (NORSE) will require induced coma with midazolam, pentobarbital or propofol in an intensive care unit (ICU) setting.^10^ In super refractory status-epilepticus, effective seizure control may not be achieved until sufficient immunosuppression is in effect. In many patients, improvement of LPDs and other EEG abnormalities may be followed by improvement in mental status. Patients may not need long-term antiseizure medications after resolution of the acute attack. The nationwide retrospective study by de Bruijn and colleagues highlighted the central role of immunosuppression in controlling AE seizures and showed that almost all surviving patients with NMDAR, LGI1 and GABA-B-R-antibody encephalitis remained seizure-free and could be weaned off seizure medications successfully after immunosuppression and resolution of brain inflammation.^6^ Antiseizure medications have several side effects and weaning should be considered in recovered patients with normal brain MRI and EEG. Due to medical, social and driving privilege implications, data beyond 5 years of follow-up and from all AE subtypes is still needed before making definitive generalised recommendations regarding the optimal duration of antiseizure mediations following the initial AE attack. Clinicians should practice caution and consider several factors when making this decision including the type of antibody, the severity of the initial presentation, MRI and EEG findings, tolerability of the antiseizure agent, and local and national epilepsy guidelines. Patients who present initially with NORSE may be at a higher risk for chronic epilepsy. In the largest case series of NORSE (all aetiologies) to date, 37% of patients later developed chronic epilepsy and 92% of survivors remained on antiseizure medications.^11^

#### Management of movement disorders

Mild movement disorders in the setting of AE do not require specific symptomatic therapy as they may improve with immunotherapy alone. Severe, dangerous or disabling movement disorders will require phenomenology-directed treatment.^12^ Severe dystonia may be treated with anticholinergics or muscle relaxants (eg, trihexyphenidyl, baclofen, respectively); myoclonus, stiff person syndromeand progressive encephalomyelitis with rigidity and myoclonus can be treated with benzodiazepines; catatonia may respond to intravenous lorazepam and/or electroconvulsive therapy although the relapse rate and cognitive impact of the latter is unknown in patients with AE.^1 12^ Severe chorea, athetosis and ballism can be treated with a cautious use of dopamine-blockers or depleters (eg, risperidone, tetrabenazine, respectively) while carefully watching for any paradoxical worsening of other involuntary movements. Dopaminergic treatment with dopamine agonists or carbidopa/levodopa may be tried in patients with acquired parkinsonism or severe akinetic-rigid syndrome.^12^

#### Management of dysautonomia

In most cases, supportive therapy with continuous monitoring in an ICU setting along with immunotherapy is all that is needed in dysautonomic patients. However, on rare occasions, symptomatic treatment with non-selective beta-blockers, alpha-2 agonists and/or acetylcholinesterase inhibitors may be required to ameliorate sympathetic overactivity. Patients with severe symptomatic postural hypotension may require midodrine, fludrocortisone or droxidopa in addition to good hydration and compressive stocking usage. Temporary pacing may be required in patients with acquired heart block or severe arrhythmias. In addition to symptomatic pharmacotherapy, patients with severe gastrointestinal dysmotility may require temporary total parenteral nutrition, and those with urinary retention often require indwelling catheters. Patients with central hypoventilation require artificial ventilation.

#### Management of sleep dysfunction

Improved sleep facilitates control over agitation, seizures and psychosis. Improving the sleep cycle is imperative in patients with AE and should be among the priorities of symptomatic therapy. The use of environmental conditioning and sleep hygiene, along with pharmacological measures such as melatonin, sedating benzodiazepines (eg, clonazepam or diazepam) and/or non-benzodiazepine hypnotics (eg, zopiclone) should be considered as appropriate for patients with AE with sleep dysfunction.^3^

### MANAGEMENT OF ASSOCIATED NEOPLASM IF PRESENT

When a paraneoplastic aetiology is confirmed, treatment of the neoplasm may result in neurological improvement or remission in some cases with or without immunotherapy.^13^ In cases associated with classical onconeuronal antibodies, tumour resection may be the intervention with the highest therapeutic benefit since neurological symptoms tend to be immunotherapy-resistant in many of those patients.^14^ In inoperable tumours, debulking surgery or palliative radiotherapy or chemotherapy may result in neurological improvement by reducing the abnormal immune drive.^15^ Of note, the Karnofsky Performance Status score may be poor due to the paraneoplastic syndrome rather than the direct effect of cancer so low scores should not hinder aggressive oncological management. Neurologists should consult with the appropriate oncology service and advocate for timely oncological intervention in order to expedite neurological recovery and prevent permanent neurological disability.

For antibodies against neuronal surface antigens in the presence of a neoplasm, AE tends to be responsive to immounomodualting therapy but tumour treatment is still necessary for neurological improvement. For example, along with immunotherapy, resection of ovarian or testicular teratoma may accelerate remission in NMDAR-antibody encephalitis.^4^ Studies have shown a germinal centre-like histology of the ovarian teratomas, with intramural NMDAR-specific B-cells that can cross the blood brain and evolve into antibody-producing intrathecal plasmablasts.^16–18^ This suggests a plausible biological basis for the observed improvement after tumour resection. The same goes for other neuronal surface antibodies associated with various benign or malignant neoplasms like AMPA-R (α-amino-3-hydroxy-5-methyl-4-isoxazolepropionic acid) and GABA/BR antibodies.

In addition to conventional antineoplastic treatments via surgical resection, chemotherapy and radiotherapy, the recent introduction of cancer-directed immune checkpoint inhibitors adds a new layer of complexity to the management of paraneoplastic AE. Although anticancer treatment usually contributes to neurological improvement, the use of ICIs is likely to trigger new paraneoplastic reactions or exacerbate pre-existing paraneoplastic AE due to the ‘unchecked’ immune response against tumour (and neuronal) antigens.^19^ Fortunately, the symptoms of paraneoplastic AE in the setting of ICIs are usually steroid-responsive.^19^ Per the recommendations of the European Society of Medical Oncology guidelines, steroids should be initiated and ICIs should be interrupted for moderate neurological side effects (grade-2) and permanently discontinued in severe cases (grade-3).^20^ The National Comprehensive Cancer Network guidelines consider meningitis and encephalitis as moderate or severe ICI neurotoxicity.^21^ If there are no other alternatives for oncological therapy, or based on patient preferences, rechallenge with ICIs may be carefully considered in selected cases after sufficient corticosteroid treatment and resolution of neurological symptoms.

### BRIDGING IMMUNOTHERAPY ON DISCHARGE

After acute treatment, it is important to avoid abrupt discontinuation of immunotherapy to prevent early recurrence.^22–24^ Therefore, a bridging strategy should be implemented followed by slow weaning or initiation of long-term immunotherapy, if indicated. A common strategy is to start oral prednisone 1–2 mg/kg/day immediately after completing acute therapy followed by a gradual taper over weeks to months overlapping with long-term immunotherapy if indicated. The rate of taper varies according to the clinical syndrome, clinical context, relapse risk, and treatment response and tolerability. However, this approach may not be suitable for patients with ongoing behavioural issues or who have contraindications to maintenance corticosteroid therapy. An alternative strategy is to give periodic intravenous methyl-prednisolone (IVMP) or intravenous immunoglobulins (IVIg) as a maintenance therapy for the same duration.^25^ If a second-line agent such as rituximab is used during the acute attack, it may serve as a bridging therapy in itself given its long-term effects.^22–24^ However, corticosteroid overlap may still be needed with the initial rituximab dose to avoid possible treatment-related relapses, in alignment with reports in patients with neuromyelitis optica spectrum disorder (NMOSD)^26^ although AE and NMOSD are substantially different conditions. When using prednisone for extended periods of time, it is important to mitigate corticosteroids toxicity by cotreatment with proton pump inhibitors, vitamin D supplements and antibiotic prophylaxis against *Pneumocystis jiroveci* pneumonia when indicated. It is also important to ensure good control of blood pressure and blood glucose while on corticosteroids.

On our AEACN survey (see online supplemental appendix 2 for details), the most popular bridging therapy was oral prednisone taper chosen by 38% of clinicians with 28% choosing to taper over months and 10% choosing to taper over days to weeks. This was followed by periodic IVIg (28%), rituximab alone or with oral prednisone (16%), and weekly or monthly IVMP (12%) including an approach of gradually increasing the intervals between infusions.

10.1136/jnnp-2020-325302.supp2 Supplementary data

### SECTION 3: LONG-TERM MANAGEMENT OF AE

Perhaps one of the most understudied aspects of AE is its long-term outpatient management following the initial attack. A major obstacle is identifying a clinician with expertise and interest in the long-term management of AE. Possible solutions include the integration of formal AE training in clinical neuroimmunology and MS fellowships or developing dedicated autoimmune neurology fellowships focusing on AE and related conditions (mirroring the limited autoimmune neurology programmes that are currently available in select institutions). Teleneurology and virtual visits may be another option to connect patients in remote areas to experts in academic centres. The long-term management of AE entails several equally important components as detailed later.

#### Interpretation of NAA panel results

Unlike acute management, the long-term management of AE is highly influenced by the presence and type of NAAs.^27^ In some cases, the results of the NAAs panel become available after the patient has been discharged although in patients with prolonged hospitalisation (eg, NMDAR-antibody encephalitis), the results become available while the patient is still hospitalised and can influence acute management. It is important to select a laboratory that uses the best available method for antibody detection. Cell-based-assay is the preferred method for neuronal surface antibodies while indirect tissue immunofluorescence and immunohistochemistry followed by Western blot confirmation is the standard for antibodies against intracellular antigens.^25^ Proper case selection for testing increases the likelihood of a positive test.^28^ Predicting scores such as the Antibody Prevalence in Epilepsy score (table 2) can help in case selection for NAAs testing factoring in the clinical presentation, cerebrospinal fluid (CSF) and MRI findings, and cancer history.^28^ A score greater than 3 predicts a high likelihood of identifying a neuronal specific antibody. Furthermore, it has been proposed that this scale could be used in the diagnostic criteria of AE in that a score greater than 3 with positive neuronal specific antibody is antibody positive AE and a score greater than 6 is probable AE.

**Table 2:** Antibody prevalence in epilepsy and encephalopathy (APE2 score)

Antibody prevalence in epilepsy and encephalopathy (APE2 score)	Value
New onset, rapidly progressive mental status changes that developed over 1–6 weeks or new onset seizure activity (within 1 year of evaluation)	(+1)
Neuropsychiatric changes; agitation, aggressiveness, emotional lability	(+1)
Autonomic dysfunction (sustained atrial tachycardia or bradycardia, orthostatic hypotension (≥20 mm Hg fall in systolic pressure or ≥ 10 mm Hg fall in diastolic pressure within 3 min of quiet standing), hyperhidrosis, persistently labile blood pressure, ventricular tachycardia, cardiac asystole or gastrointestinal dysmotility)	(+1)
Viral prodrome (rhinorrhoea, sore throat, low-grade fever) to be scored in the absence of underlying systemic malignancy within 5 years of neurological symptom onset	(+2)
Faciobrachial dystonic seizures	(+3)
Facial dyskinesias, to be scored in the absence of faciobrachial dystonic seizures	(+2)
Seizure refractory to at least to two antiseizure medications	(+2)
CSF findings consistent with inflammation (elevated CSF protein >50 mg/dL and/or lymphocytic pleocytosis >5 cells/µL, if the total number of CSF RBC is <1000 cells/µL)	(+2)
Brain MRI suggesting encephalitis (T2/FLAIR hyperintensity restricted to one or both medial temporal lobes, or multifocal in grey matter, white matter or both compatible with demyelination or inflammation)	(+2)
Systemic cancer diagnosed within 5 years of neurological symptom onset (excluding cutaneous squamous cell carcinoma, basal cell carcinoma, brain tumour, cancer with brain metastasis)	(+2)
	Total (max: 18)

Adapted with permission from Dubey *et al*.^28^

CSF, cerebrospinal fluid.

When interpreting the NAAs panel, four possible results may be encountered. The first possibility is positivity for an antibody against intracellular antigens. These antibodies are highly specific and usually predictive of a paraneoplastic aetiology and a recognisable paraneoplastic syndrome in most cases especially those with typical clinical phenotype.^29^ A recent study evaluating the diagnostic yield of commercial onconeuronal antibodies in France found a low cancer predictability rate in a cohort of patients with frequent non-classical clinical presentations and questionable laboratory results (confirmed by another technique in only 30% of cases).^30^ The second possibility is positivity for one of the highly clinically relevant antibodies against neuronal surface antigens such as NMDAR or LGI1-antibodies. These antibodies are highly specific with reasonable positive predictive value for neurological autoimmunity and are known to be clinically relevant when present in the proper clinical setting.^27^ They can be associated with idiopathic or paraneoplastic forms of AE. Paraneoplastic cases are most frequently associated with NMDAR, AMPAR and GABA/BR antibodies.^25 27^ The third possibility is positivity for an antibody against neuronal surface antigens with limited clinical relevance such as VGCC, non-LGI1 non-CASPR2 ‘double negative’ VGKC and ganglionic AChR antibodies (table 1, part-1). These antibodies may or may not be relevant to the patient’s presentation depending on the clinical picture, and their presence should not preclude thorough exclusion of other potential causes of the neurological presentation.^31–33^ The antibody level (for some antibodies like GAD65-antibody), clinical presentation, disease course, CSF findings and smoking or cancer history are factors that can be used to determine the clinical relevance of the positive antibody.^33–35^ The NAAs confidence scale is one suggested tool to increase the confidence in the clinical relevance of these less specific antibodies (table 3).^33^

**Table 3:** Neuronal Autoantibody Confidence Scale*

Clinical/laboratory factor	Score
Ab against intracellular antigen (or high clinical relevance surface antibody)	1
Movement disorder and/or stiff person syndrome	1
Cancer and/or smoking history	1
Inflammatory CSF (either high cell count, IgG index and/or positive OCBs)	1
Serum hyponatraemia	1
Chronic course (>3 months)†	−1
Total	Maximum=5 Minimum=−1

Modified from Abboud *et al.* 33

*Based on a study in patients tested for the original Mayo Clinic Paraneoplastic panel not the Autoimmune Encephalitis Panel.

†Although chronic course is rare in autoimmune encephalitis, patients with leucine-rich glioma inactivated-1, CASPR2 and IgLON5-antibodies can have a chronic course.

Ab, antibody; CSF, cerebrospinal fluid; OCBs, oligoclonal bands.

This scale has a 77% sensitivity, 94% specificity, 87% positive predictive value and 89% negative predictive value for clinical relevance of the positive NAAs. If the score is greater than 1, it is likely that the antibody is clinically relevant. Conversely, if the score is less than 1, it is likely that the antibody is clinically irrelevant, whereas a score of 1 is not predictive. If an alternative diagnosis was found during workup (eg, neurosarcoidosis, nutritional deficiency) then the positivity of one of these less specific antibodies should be considered a clinically irrelevant result not necessitating repeat cancer screening or addition or change of immunotherapy.^33 34^ It is to be noted that the clinical relevance of some of these antibodies is higher for peripheral neurological disorders as in the case of VGCC antibody with Lambert-Eaton myasthenic syndrome, and ganglionic AChR antibody with autoimmune autonomic ganglionopathy. Therefore, it is important to always correlate the clinical presentation to the positive antibody and question the clinical relevance of the test result if there is clinical-serological discordance. More recent NAAs panels emphasise the importance of clinical correlation and are based on clinical presentation (movement disorders vs epilepsy vs encephalopathy, etc) as opposed to aetiology (paraneoplastic vs idiopathic). As our knowledge and understanding of autoimmune neurology expands, antibodies with limited clinical relevance are expected to become obsolete or limited to specific panels.

**Figure 1:**
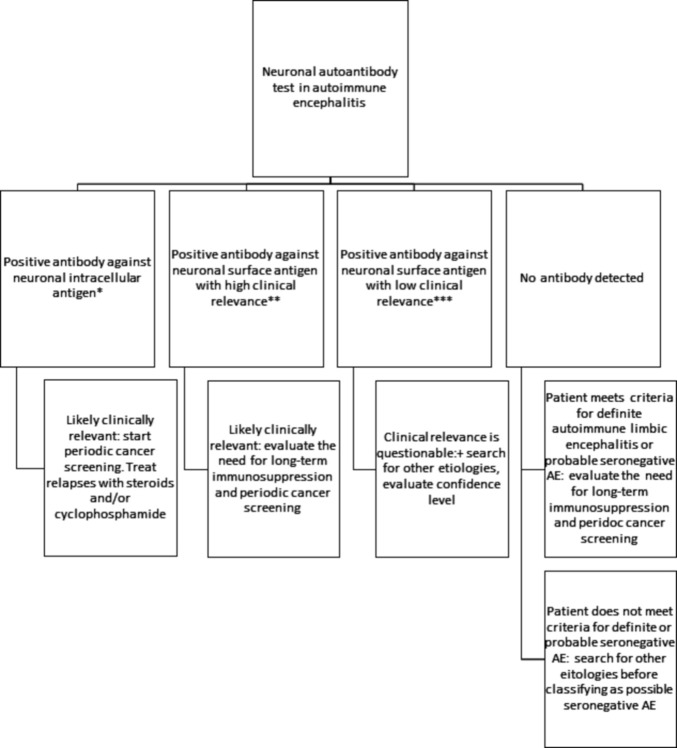
Interpretation of the neuronal autoantibody panel. *Anti-Hu (ANNA-1), anti-Ri (ANNA-2), ANNA-3, anti-SOX1 (AGNA), anti-amphiphysin, anti-CRMP-5 (anti-CV2), anti-Yo (PCA-1), PCA-2, high-titre anti-GAD65. **Anti-NMDA-R, anti-LGI1, anti-CASPR2, anti-AMPA-R, anti-GABA-A/B, PCA-Tr, anti-DPPX, anti-mGluR1, anti-mGluR2, anti-mGluR5, anti-IgLON5, anti-AQP4, anti-MOG. ***Non-LGI1 non-CASPR2 anti-VGKC, anti-P/Q VGCC, anti-N VGCC, Ach-b, Ach-M, Ach-G, Striational. Low-titre anti-GAD65 is an antibody against cytoplasmic antigen but is of questionable clinical significance. Adapted with permission from George *et al*.^40^

The fourth possibility is negativity for all commercially available antibodies. In that situation, it is important to determine whether the patient meets criteria for definite autoimmune limbic encephalitis or probable seronegative AE (online supplemental boxes 1 and 2).^27^ Patients with probable or definite seronegative AE should be tested for novel antibodies in research neuroimmunology laboratories if access to one is available (examples include Mayo Clinic, Pennsylvania, Oxford, Erasmus and Barcelona universities). If a patient was treated empirically for possible AE in the acute setting but tested negative for NAAs and did not meet criteria for definite or probable seronegative AE, it is very important that the diagnosis is challenged and workup for other potential diagnoses is initiated or repeated, especially if the response to immunotherapy was limited (figure 1).

10.1136/jnnp-2020-325302.supp3 Supplementary data

10.1136/jnnp-2020-325302.supp4 Supplementary data

#### Determining the need for long-term immunosuppression

As mentioned previously, it is important to initiate bridging immunosuppression after acute therapy in the hospital followed by a gradual taper. The more difficult task is selecting patients for long-term immunosuppression. The recurrence rate is highest in conditions associated with clinically relevant neuronal surface antibodies and much lower in conditions associated with antibodies against intracellular antigens, which tend to follow a relentless progressive course rather than a relapsing one but may remit after cancer treatment in some patients.^25 27^ Determining what constitutes a recurrence is itself a difficult task. Fluctuation of cognition, breakthrough seizures and other transient worsening of residual symptoms after the initial attack are common and do not necessarily represent a recurrence of the autoimmune inflammation. In some AE types, relapses tend to be phenotypically identical to the initial attack as in LGI1-antbiody encephalitis^36^ or similar but milder in severity as in NMDAR-antibody encephalitis.^36^ In other AE subtypes, relapses can present differently from the initial attack, as is the case of CASPR2-antibody encephalitis.^37^ In all cases, getting supportive objective information from MRI, EEG and/or CSF can help confirm a true relapse. Recurrence rates in AE associated with neuronal surface antibodies range from 10% to 35% based on retrospective observational studies but these rates are confounded by short follow-up periods in most of the reported case series and review articles.^13 22 24 38^ On the other hand, suspecting AE and testing the antibody panel may sometimes happen only after a relapse of encephalopathy so the true rates of monophasic disease may also be underestimated.^38^ The recurrence rate is unknown in seronegative AE.

With this uncertainty and the low recurrence rates in seropositive cases, it is difficult to justify prolonged immunosuppression though in some cases a few to several years of maintenance immunosuppression may be indicated. Decisions regarding long-term immunosuppression should take in consideration published relapse rates for each specific clinical syndrome as well as severity of the initial attack and individual risks related to immunosuppression. Relapse rates and the value of long-term immunosuppression are among the key areas in need for further future research. In the meantime, any decision regarding maintenance immunosuppression in patients with AE should carefully weigh the risks versus potential benefits and incorporate evolving data about relapse risk for each specific clinical syndrome. Patients who experience a definite clinical relapse based on high clinical suspicion and supported by objective evidence of ancillary tests (eg, MRI or EEG) should start long-term immunosuppression after relapse treatment.^23^ Although azathioprine and mycophenolate mofetil (MMF) have been used in this setting, the use of rituximab may have the added benefit of a potentially faster onset of action (second-line acute therapy) and less carcinogenic potential with prolonged use compared with other agents.^23 24^ Rituximab can be used as both a second-line agent for acute immunosuppression and as a long-term immunosuppressant for recurrent cases. Rituximab, however, does not deplete the antibody-secreting cells which are typically CD20-negative. In these conditions, rituximab may work by deleting the antigen-specific memory B-cell populations and hence preventing the formation of new plasmablasts which secrete the pathogenic antibodies.^17^ The use of other B-cell therapies (eg, humanised anti-CD20 and anti-CD19 monoclonal antibodies) may be worth exploring in future research. Overlapping with oral corticosteroids is needed for 3–6 months when using azathioprine or MMF due to their delayed onset of action.

On our AEACN survey, in response to a question in the check-all-that-apply format, 70% of responders indicated they would start long-term immunosuppression in AE associated with antibodies against neuronal surface antigens after a second attack while 50% indicated they would start after the first attack. As for seronegative AE, 61% indicated they would start long-term immunosuppression after a second attack and only 10.4% indicated they would start after the first attack. However, these generalised survey results should be treated with caution since clinicians’ practice is influenced by the specific AE subtype they see most frequently. In addition, many clinical specifics influence the decision regarding long-term immunosuppression as mentioned earlier.

For patients with antibodies against intracellular antigens in whom the associated tumour has been treated, shorter bridging therapy may be considered. This is because recurrence rates are low after tumour treatment and since the response to immunotherapy is generally limited in those patients.^23 24 29^ In patients with antibodies against intracellular antigens in whom no tumour was found, a shorter course of bridging therapy is also advisable especially if they have not had a robust response to acute immunotherapy since prolonged immunosuppression may increase the risk of progression of the presumed underlying tumour. Long-term immunosuppression should generally be used with caution in those patients for the same reasons. This concept was reflected in the AEACN survey results as only 29% of responders indicated they would start long-term immunosuppression after treatment of the coexisting tumour for AE associated with antibodies against intracellular antigens and only 46% indicated they would start long-term immunosuppression if a tumour was not found. Patients with ongoing progression of neurological disability may be selected for immunosuppression with careful and frequent cancer screening.

On the AEACN survey, the most popular choice for long-term immunosuppression for relapsing AE was rituximab chosen by 46% of responders, followed by azathioprine (15%), MMF (12%), maintenance corticosteroids (6%) and maintenance IVIg (4%). Some clinicians (12%) indicated that their choice of the long-term immunosuppressive agent depends on the antibody type with rituximab being preferred for antibodies against neuronal surface antigens (humoral autoimmunity) and other agents such as azathioprine or MMF preferred for antibodies against intracellular antigens and for seronegative AE (for presumed cellular autoimmunity). Some responders stressed the importance of patients’ comorbidities and preferences in making this decision.

The optimal duration of maintenance therapy in relapsing forms of AE is unknown but published empiric approaches suggest initial maintenance period of 3 years followed by re-evaluation and attempt at withdrawal of immunosuppression.^22–25^ This suggested duration of long-term immunotherapy is arbitrary and not evidence-based. Retrospective studies in NMDAR-antibody encephalitis showed a small rate of recurrence within a 2-year duration but patients who received second-line immunotherapy (predominantly rituximab) had lower recurrence rates.^22^ Patients who have more than one relapse while on immunosuppression or while being weaned should be considered for extended immunosuppression.^23^ On the AEACN survey, the most popular choice for the duration of long-term immunosuppression in relapsing AE was 3 years selected by 44% of responders followed by 2 years (19%), 1 year (13%), lifelong (7%) and 6 months (3%). Of note, 13% of survey responders indicated that the duration of immunosuppression would depend on multiple factors including severity of prior attacks, tolerability of the immunosuppressive agents, antibody type and patient’s comorbidities and cancer risk.

The best long-term preventive therapy for relapsing AE depends on the specific immunopathology of each AE subtype. The current empiric approaches are expected to improve as the specific pathogenic mechanism of each serological and/or clinical AE subtype is refined. A tailored approach with more selective immunomodulation to each specific syndrome will likely improve outcomes and limit unnecessary side effects. The value of non-cell-depleting immunotherapies (eg, complement or cytokine inhibitors) is yet to be fully explored in the long-term management of AE. The use of interleukein-6 inhibitors as second-line rescue therapy has already been discussed in part-1 but their use as maintenance therapy for recurrent AE is yet to be evaluated. The rarity of individual AE subtypes hinder large-scale clinical trials but this can possibly be overcome through international multicentre collaborations similar to the recent NMOSD trials. Consolidation of AE subtypes with similar pathogenic mechanisms could be considered to facilitate recruitment and expedite the advancement of evidence-based medicine in AE.

#### Determining the need for and frequency of periodic cancer screening

**Figure 2:**
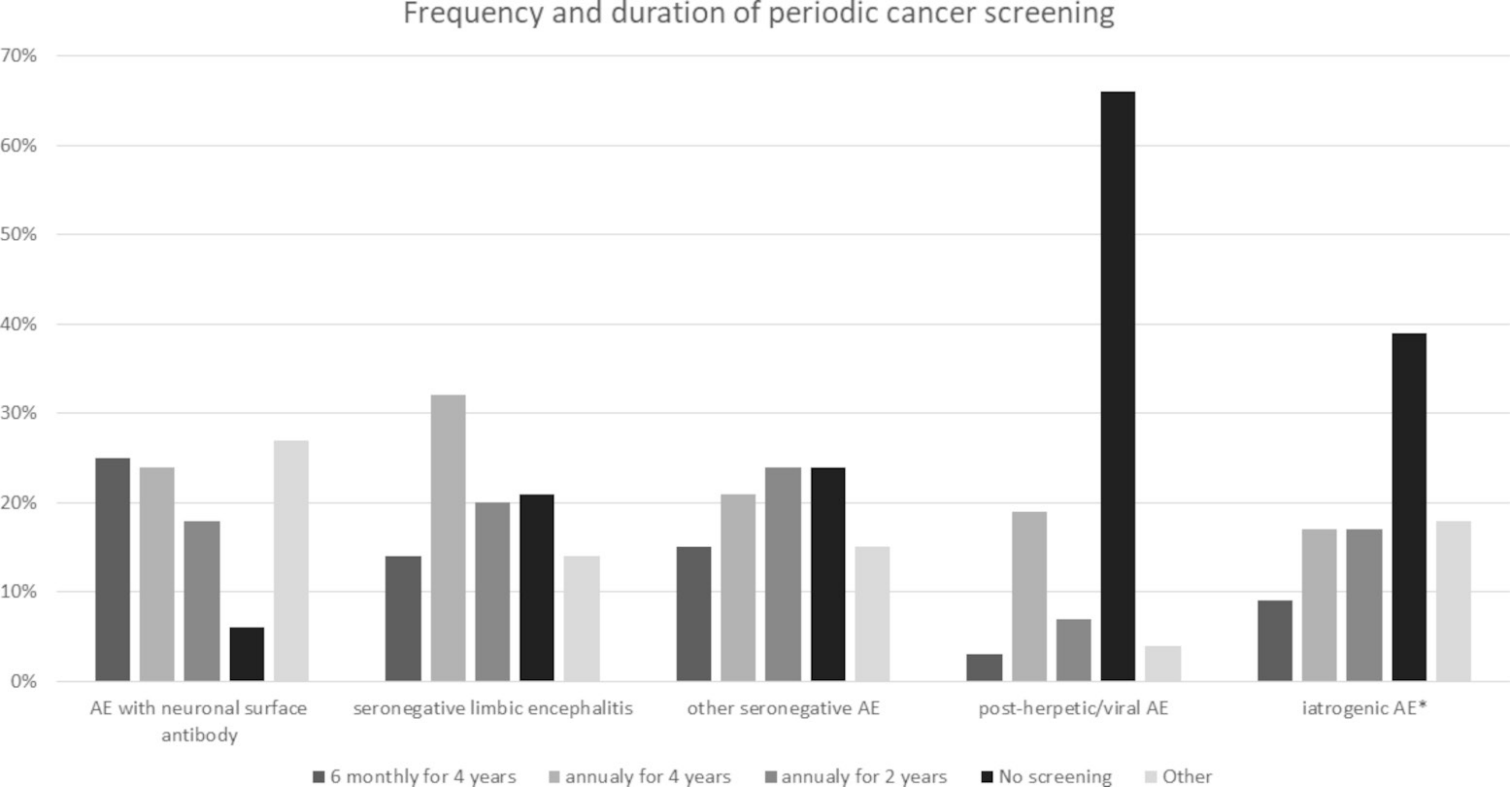
Autoimmune Encephalitis Alliance Clinicians Network survey results for periodic cancer screening. AE, autoimmune encephalitis. *Excluding immune checkpoint inhibitors.

Initial cancer screening should be considered for most adult patients with AE at the time of presentation and at the time of any definite relapse.^39^ In patients in whom a tumour was found and treated, recommendations for periodic screening are dictated by established guidelines for each cancer type. In patients in whom a tumour is not found initially, periodic tumour screening every 6–12 months for an average of 4 years should be considered for patients with antibodies against intracellular antigens given their strong association with tumours.^39^ As for antibodies against neuronal surface antigens, tumour association is less frequent and is variable from one antibody to another. There is currently no clear guidelines for the optimal frequency and duration for cancer screening in adult patients with AE with antibodies against neuronal surface antigens, which can understandably vary depending on the specific antibody. The importance of early tumour detection should be weighed against the risks of frequent and prolonged cancer screening including increased cost and the potential for incidental findings and subsequent unnecessary investigations or interventions. On our AEACN survey (figure 2), the majority of responders (49%) opted to cancer screening for 4 years in those patients with half the responders choosing semiannual screening and half choosing annual screening during that period. Screening yearly for 2 years was chosen by 18% of responders while only 6% indicated that no periodic cancer screen is necessary after the initial screen. Some clinicians (18%) indicated that the frequency and duration of cancer screening must be tailored according to published rates of cancer association for each specific antibody. However, it should also be noted that many patients with these conditions would never have a tumour discovered.

The value of periodic cancer screening in patients with seronegative AE is unknown but should be considered in patients with relapsing disease and those with definite limbic encephalitis in whom the connection to cancer is expected to be higher than other neuroanatomical variants.^29 39^ On the AEACN survey, 46% of responders indicated they would perform cancer screening every 6–12 months for 4 years in patients with seronegative limbic encephalitis while 20% chose yearly screening for 2 years and 21% indicated that no periodic cancer screening is necessary after the initial screen. As for other seronegative neuroanatomical variants (eg, cortical, brainstem), fewer clinicians felt the need to screen patients for 4 years (36%) than in the case of limbic encephalitis and relatively more clinicians chose screening for 2 years (24%) or no screening (24%). Some clinicians (10%) indicated that the frequency and duration of cancer screening in seronegative AE would depend on each patient’s demographics and social habits (eg, age, smoking, etc).

In patients with AE in the setting of ICI cancer treatment, cancer monitoring will be dictated by the oncologist according to established guidelines for each cancer type. When AE occurs in the setting of other immunomodulating therapies (eg, TNF-alpha inhibitors, daclizumab) or other well-known triggers (eg, post-herpetic), periodic cancer screening may not be as imperative since a paraneoplastic aetiology is less likely in the presence of an established trigger. On our AEACN survey, this concept was reflected in the answers addressing cancer screening following post-herpetic AE as 65% of responders indicated that there is no need for periodic screening following the initial screen. However, in iatrogenic AE in the setting of immumodulating therapies other than ICIs, only 39.3% of responders opted not to perform periodic cancer screening after the initial one indicating less confidence in the aetiological relationship between these agents and AE development especially in patients with cancer risk factors. Nevertheless, most clinicians selected less stringent cancer screening protocols in patients with iatrogenic AE (only 25% recommended cancer screening for a duration of 4 years).

Whole body FDG-PET is a single test that may be used for periodic screening in addition to recommended age appropriate screening tests (eg, mammograms, colonoscopy).^39^ An initial first-line screening study (eg, CT) may be required prior to approval of FDG-PET, although approval policies vary by insurer. FDG-PET can detect tumours that are missed by CT making it a higher yield test in paraneoplastic conditions since associated tumours are usually in early development.^39^ Medical insurance providers should allow FDG-PET coverage in patients with paraneoplastic syndromes and/or positive NAAs as discussed in part-1. FDG-PET is not ideal for seminoma/teratoma detection so periodic pelvic/scrotal ultrasound should be considered in case of AE serological or phenotypical subtypes suggestive of these tumours (eg, anti-NMDR or anti-Ma2 encephalitis or their phenotypes). A more targeted periodic cancer screening can also be considered for certain antibodies with specific cancer associations (eg, pelvic ultrasound and mammogram/breast MRI for anti-Yo antibody).

#### Physical and neuropsychological rehabilitation

Patients with ataxia, spasticity and other mobility issues may benefit from physical therapy and neurorehabilitation. More importantly, patients with short-term memory impairment and other cognitive deficits should undergo neuropsychological evaluation to identify those in need for neuropsychological rehabilitation programmes. The value of cognitive and neuropsychological rehabilitation in AE has not been investigated in a systematic manner but clinical experience supports a pivotal role in recovery after the acute phase. Response to neuropsychological rehabilitation may vary according to patient’s age, comorbidities and extent/location of permanent brain damage if any. It is unknown if antibody type influences responsiveness to neuropsychological rehabilitation. Studies on cognitive outcomes of AE and the role of neuropsychological rehabilitation is among the most pressing needs in AE research. Some patients may require modification of their house or workplace. Many patients may need formal functional capacity evaluations to determine their ability to go back to the workforce, and most will need aggressive management of vascular risk factors and promotion of healthy lifestyle to avoid further cognitive decline.

### DISCUSSION AND SUMMARY

In this two-part project, we analysed each step in AE management in a real-life chronological order that covers the first neurological presentation, diagnostic workup, acute management, bridging therapy, and long-term management and monitoring. We focused on practical management questions and used published research and expert opinion to provide broad recommendations to clinicians. We understand that AE is a heterogeneous disease and that treatment strategies may differ from one antibody-related syndrome and/or one clinical subtype to another. However, individual AE syndromes are rare and information on the specific antibody is usually lacking at the time of presentation. This makes it necessary to establish a common general approach to AE to guide initial management until the specific antibody is revealed. Moreover, many cases of AE are not linked to any of the commercially available antibodies, which adds to the importance of having a common approach. In addition, many AE syndromes have common clinical and pathogenic features making standardisation of certain aspects of both acute and long-term management possible for some of these syndromes.

A major limitation to our survey questionnaire is generalisation. When addressing a diverse clinical entity like AE with a wide spectrum of clinical phenotypes and patient demographics/comorbidities, it is difficult to develop specific questions for every possible clinical scenario. Therefore, our recommendations may not be suitable for all patients and clinicians will still need to make individual decisions based on each patient’s unique circumstances.

Our AEACN survey results highlight the diversity of practice across institutions when it comes to AE management and emphasise the need for development of standards of care. Although no consensus was reached for most of the survey questions, the survey results showed which approaches are most popular among AE clinicians and which steps in AE management are most divisive and therefore require more research. More formal consensus techniques like the Delphi method were not implemented to avoid misinterpretation of our recommendations as firm treatment guidelines. A major goal of this paper was to showcase both agreements and disagreements in AE management in order to inform future observational and interventional studies. In this evolving field, presenting firm consensus guidelines in the absence of strong scientific evidence can have a negative impact on future research efforts. On the other hand, translating practice patterns into management recommendations remains a major limitation to this paper. However, the recommendations did not rely solely on survey results and incorporated available evidence from several AE subtypes and related immune-mediated disorders.

The inclusion of multiple subspecialties and several countries in the survey enriched the results and made our recommendations applicable to a larger audience. However, this diversity in specialty and geographical locations inevitably introduced a degree of responder bias given the difference in practice per specialty (eg, paediatric vs adult neurologists) and location (eg, some therapeutic and diagnostic interventions are not readily available in some countries/institutions).

Our recommendations are meant to serve as a general guidance for clinicians until better quality evidence becomes available for each AE subtype and are expected to evolve over time as more data emerge in the future. A summary of the recommendations for acute management was presented after part-1. Box 1 includes a summary of the recommendations for long-term management.

Box 1 **Best practice recommendations summary for long-term management of autoimmune encephalitis**

1. Positive antibody against intracellular antigen (classical onconeuronal antigens) and typical clinical picture: refer to oncology for treatment and surveillance of tumour if one was found. If no tumour was found, initiate semiannual to annual cancer screening for at least 4 years. Treat neurological relapses with intravenous methyl-prednisolone and/or cyclophosphamide as necessary but avoid long-term immunosuppression.
2. Positive antibody against neuronal surface antigen with high clinical relevance and typical clinical picture: consider periodic tumour screening based on the type of antibody and each patient’s cancer risk factors. Some neuronal surface antibodies with higher rates of tumour association may require more frequent screening as in gamma-Aminobutyric acid-B receptor (GABABR)-antibody encephalitis and some may require less frequent screening as in leucine-rich glioma inactivated-1-antibody encephalitis. Consider initiating at least annual cancer screening for an average of 2–4 years based on antibody type. A more selective screening approach could be considered for antibodies with specific tumour associations. Consider long-term immunosuppression preferably with rituximab (based on presumed antibody-mediated immunity and on N-methyl-D-aspartate receptor (NMDAR)-antibody encephalitis studies) after a second attack. May consider starting long-term immunosuppression after the first attack in patients with severe initial presentation or risk factors for relapse (eg, persistently positive oligoclonal bands). Overlap with short-term oral corticosteroids after initiation of long-term agent. The duration of long-term immunosuppression depends on relapse rate, relapse severity and tolerability of the immunosuppressive agent.
3. Positive antibody against neuronal surface antigen with low clinical relevance to the clinical presentation: evaluate confidence in the clinical relevance of the positive antibody based on clinical and ancillary data. Evaluate for alternative aetiologies. If the diagnosis of autoimmune encephalitis (AE) is felt to be probable and no other aetiology found then follow recommendation 2.
4. Seronegative AE: confirm the diagnosis according to published criteria and exclude alternative causes. May consider initiating annual cancer screening for an average of 4 years for seronegative definite autoimmune limbic encephalitis and may consider periodic screening for an average of 2 years for all other neuroanatomical variants. Start long-term immunosuppression with rituximab, mycophenolate mofetil or azathioprine after a second attack. Overlap with short-term corticosteroids after initiation of long-term agent. The duration of long-term immunosuppression depends on relapse rate, relapse severity and tolerability of the immunosuppressive agent. Recommendations for seronegative AE are particularly anecdotal and more research is needed for this subtype of AE.
5. For all AE subtypes: treat residual symptoms including seizures, movement disorders, psychiatric symptoms, spasticity, sleep dysfunction and dysautonomia. Also start de-escalation of symptomatic medications when appropriate. Start physical, occupational and speech therapy depending on residual deficits. Strongly consider neuropsychological rehabilitation although the value behind this intervention is in need for further research to establish scientific evidence.
