# Supplementary material for: Autoimmune encephalitis: proposed recommendations for symptomatic and long-term management
Source: J Neurol Neurosurg Psychiatry. 2021 Mar 1;92(8):897–907. doi: 10.1136/jnnp-2020-325302 (PMC8292591; doi:10.1136/jnnp-2020-325302)
Supplement: Supplementary data [file jnnp-2020-325302supp001.pdf]

Appendix 1: List of neuronal auto-antibodies

A- Antibodies against intracellular antigens (classical onconeuronal antibodies):

1- Anti-Neuronal Nuclear Ab, Type 1 (ANNA1 or anti-Hu)
2- Anti-Neuronal Nuclear Ab, Type 2 (ANN2 or anti-Ri)
3- Anti-Neuronal Nuclear Ab, Type 3 (ANNA3)
4- Anti-Glial Nuclear Ab, Type 1 (AGNA1)
5- Purkinje Cell Cytoplasmic Ab Type 1 (PCA1 or anti-Yo)
6- Purkinje Cell Cytoplasmic Ab Type 2 (PCA2; MAP1b-IgG)
7- Purkinje Cell Cytoplasmic Ab Type Tr (PCATR)
8- Amphiphysin Ab (AMPH)
9- Collapsin response mediator protein 5 (CRMP-5-IgG or anti-CV2).
10- Anti-Ma1/Ma2 (or anti-Ta)
11- kelch-like protein 11 Ab (KLHL11)
12- Neuronal intermediate filament and internexin Ab (NIF)
13- Adenylate kinase 5 Ab (anti-AK5)
14- Glutamic acid decarboxylase 65 Ab (GAD65): Not considered a classical onconeruonal antibody and is associated with clinical features similar to antibodies against surface antigens.
15- Glial fibrillary acidic protein Ab (GFAP): antibody against astrocyte GFAP. Not considered a classical onconeruonal antibody and is associated with clinical features similar to antibodies against surface antigens.

B- Antibodies against neuromuscular and ganglionic synaptic antigens:

1- P/Q-Type Calcium Channel Ab (PQ-VGCC)
2- N-Type Calcium Channel Ab (N-VGCC)
3- Acetyl Choline Receptor (Muscle) Binding Ab (AChR-B)
4- Acetyl Choline Receptor (Muscle) Modulating Ab (AChR-M)
5- Acetyl Choline Receptor Ganglionic Neuronal Ab (ganglioinic AChR)
6- Striational (Muscle) antibodies (STR).

C- Antibodies against surface antigens:

1- N-Methyl D-Aspartate Receptor Ab (NMDAR)
2- α-amino-3-hydroxy-5-methyl-4-isoxazolepropionic acid receptor Ab (AMPAR)
3- Gamma-Amino butyric acid A Receptor Ab (GABA-A-R)
4- Gamma-Amino butyric acid B Receptor Ab (GABA-B-R)
5- Neuronal voltage-gated potassium channel Ab (VGKC)
6- Leucine-rich glioma inactivated Ab (LGI-1): part of the VGKC receptor complex
7- Contactin-associated protein-like 2 Ab (CASPR2): part of the VGKC receptor complex.
8- Dipeptidyl-peptidase-like protein 6 Ab (DPPX)
9- Metabotropic glutamate receptor 1 Ab (mGluR1) and anti-Homer-3
10- Metabotropic glutamate receptor 5 Ab (mGluR5)
11- Glycine receptor Ab.
12- Dopamine receptor 1 Ab (D1)
13- Dopamine receptor 2 Ab (D2)
14- IgLON5 Ab
15- Neurexin-3α Ab
16- Aquaporin 4 water channel (Astrocyte) Ab (AQP4)

17- Myelin oligodendrocyte glycoprotein (Oligodendrocyte) Ab (MOG)
