# Supplementary material for: Autoimmune encephalitis: proposed recommendations for symptomatic and long-term management
Source: J Neurol Neurosurg Psychiatry. 2021 Mar 1;92(8):897–907. doi: 10.1136/jnnp-2020-325302 (PMC8292591; doi:10.1136/jnnp-2020-325302)
Supplement: Supplementary data [file jnnp-2020-325302supp002.pdf]

Q1 Please provide your information

Answered: 68 Skipped: 0

ANSWER CHOICES	RESPONSES	
Name	100.00%	68
Institution	100.00%	68
Title	98.53%	67
Address 2	0.00%	0
City/Town	0.00%	0
State/Province	0.00%	0
ZIP/Postal Code	0.00%	0
Country	0.00%	0
Email Address	98.53%	67
Phone Number	0.00%	0

Abboud H, *et al. J Neurol Neurosurg Psychiatry* 2021; 92:897–907. doi: 10.1136/jnnp-2020-325302

Q2 What is your specialty (check all that apply)?

Answered: 68 Skipped: 0

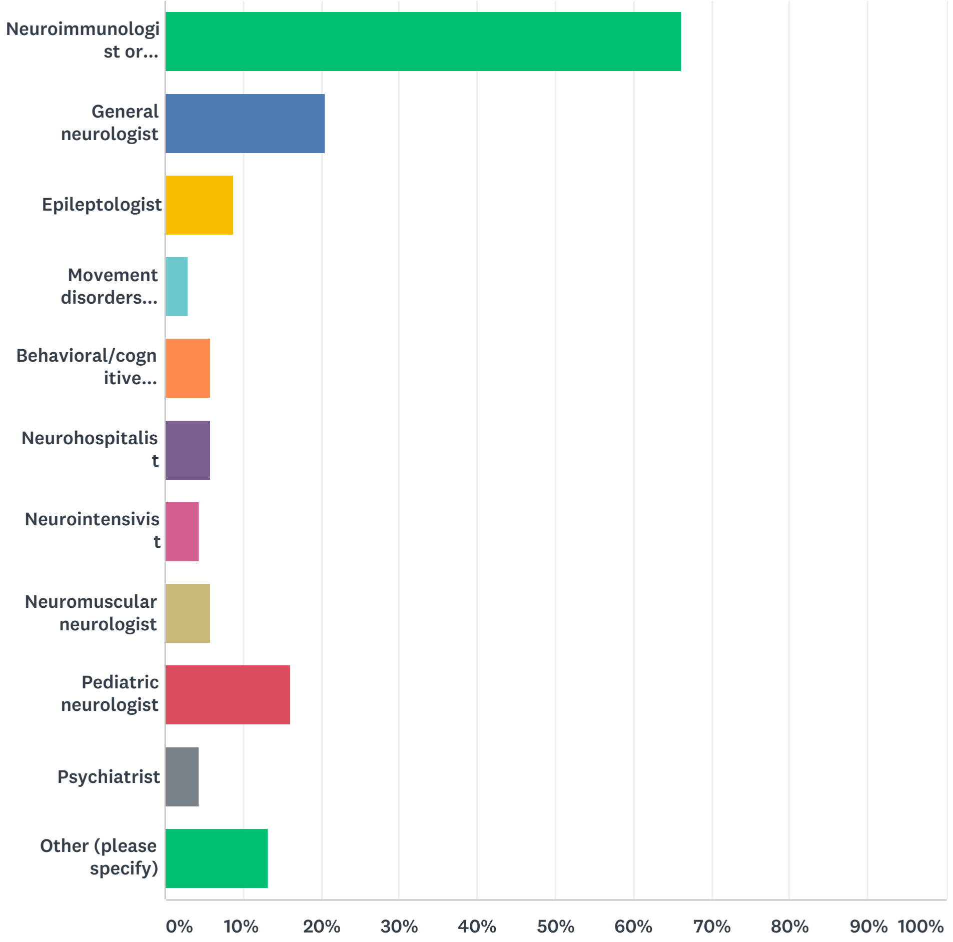

ANSWER CHOICES	RESPONSES	
Neuroimmunologist or autoimmune neurologist	66.18%	45
General neurologist	20.59%	14
Epileptologist	8.82%	6
Movement disorders specialist	2.94%	2
Behavioral/cognitive neurologist	5.88%	4
Neurohospitalist	5.88%	4
Neurointensivist	4.41%	3
Neuromuscular neurologist	5.88%	4
Pediatric neurologist	16.18%	11
Psychiatrist	4.41%	3

BMJ Publishing Group Limited (BMJ) disclaims all liability and responsibility arising from any reliance placed on this supplemental material which has been supplied by the author(s)

Other (please specify)	13.24%	9
Total Respondents: 68		

Q3 Do you want to be included in the affiliated authors on the paper?

Answered: 68 Skipped: 0

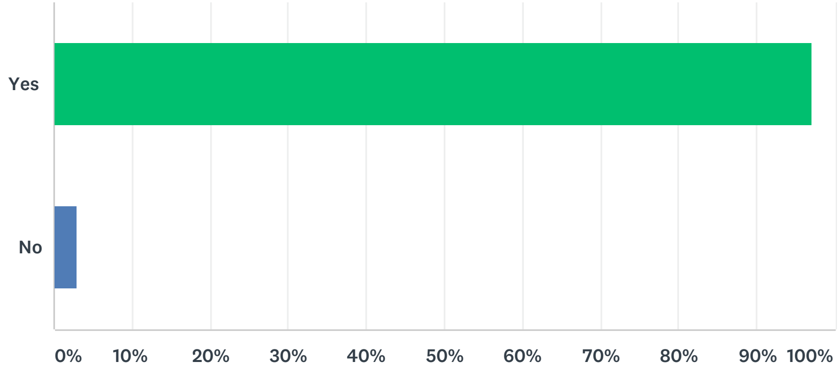

ANSWER CHOICES	RESPONSES	
Yes	97.06%	66
No	2.94%	2
TOTAL		68

Q4 Do you want to review and edit the initial manuscript when ready?

Answered: 68 Skipped: 0

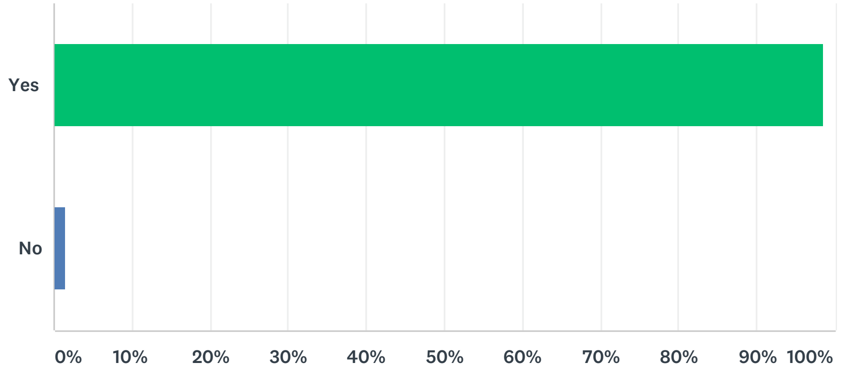

ANSWER CHOICES	RESPONSES	
Yes	98.53%	67
No	1.47%	1
TOTAL		68

Abboud H, *et al. J Neurol Neurosurg Psychiatry* 2021; 92:897–907. doi: 10.1136/jnnp-2020-325302

Q5 For acute treatment of AE, which first line therapy would you choose in a previously healthy patient?

Answered: 68 Skipped: 0

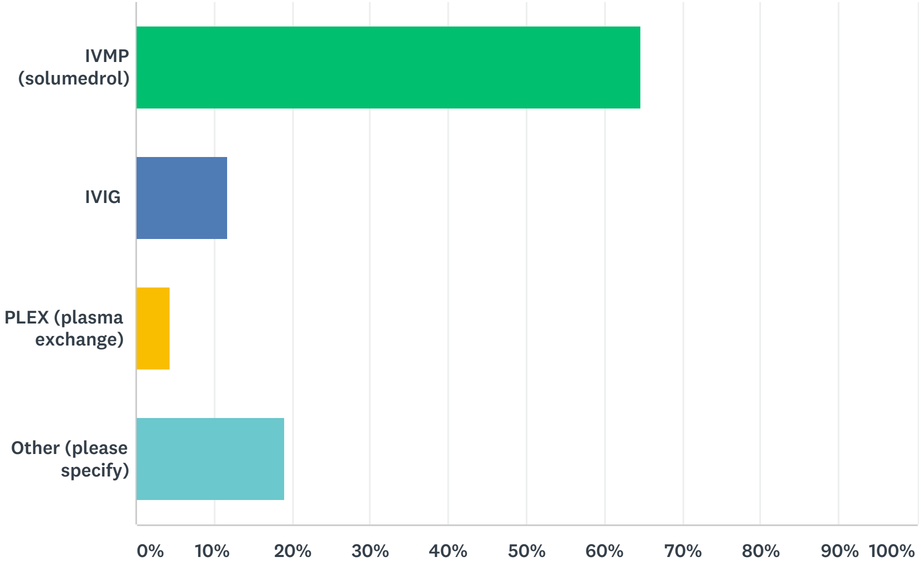

ANSWER CHOICES	RESPONSES	
IVMP (solumedrol)	64.71%	44
IVIG	11.76%	8
PLEX (plasma exchange)	4.41%	3
Other (please specify)	19.12%	13
TOTAL		68

Q6 For acute treatment of AE, which first line therapy would you choose in a patient with uncontrolled diabetes and/or hypertension?

Answered: 68 Skipped: 0

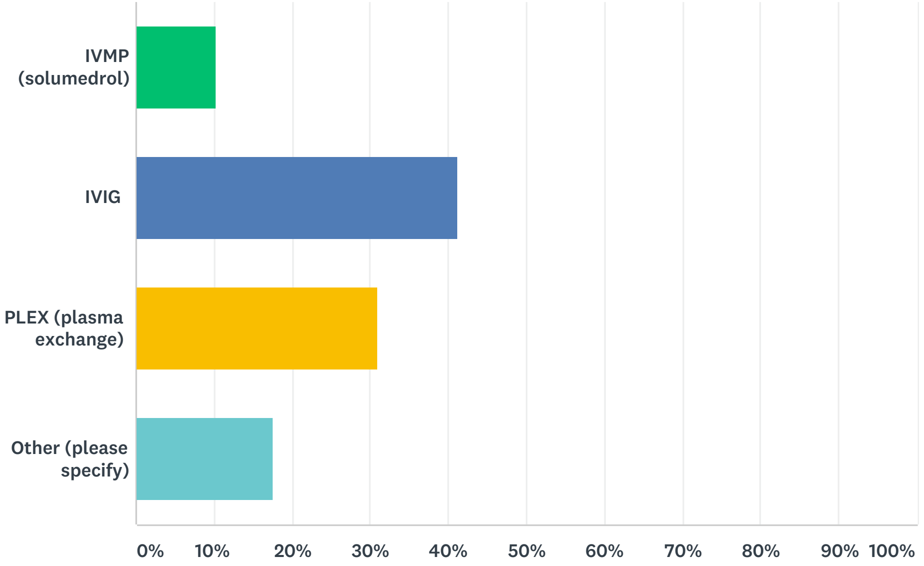

ANSWER CHOICES	RESPONSES	
IVMP (solumedrol)	10.29%	7
IVIG	41.18%	28
PLEX (plasma exchange)	30.88%	21
Other (please specify)	17.65%	12
TOTAL		68

Abboud H, *et al. J Neurol Neurosurg Psychiatry* 2021; 92:897–907. doi: 10.1136/jnnp-2020-325302

Q7 For acute treatment of AE, which first line therapy would you choose in a patient with a clinical picture suggestive of NMDAR encephalitis?

Answered: 68 Skipped: 0

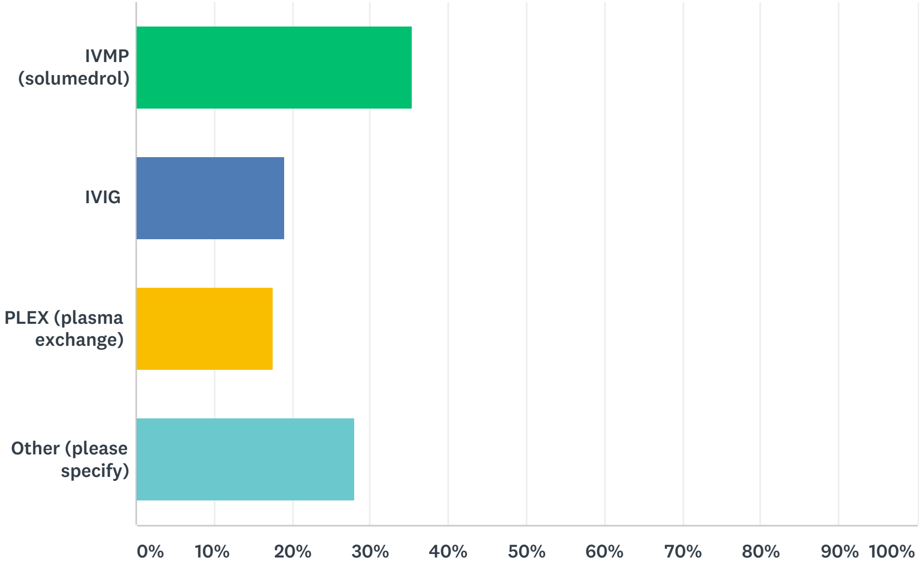

ANSWER CHOICES	RESPONSES	
IVMP (solumedrol)	35.29%	24
IVIG	19.12%	13
PLEX (plasma exchange)	17.65%	12
Other (please specify)	27.94%	19
TOTAL		68

Q8 For acute treatment of AE, which first line therapy would you choose in a patient presenting with faciobrachial dystonic seizures suggestive of anti-LGI1 encephalitis?

Answered: 67 Skipped: 1

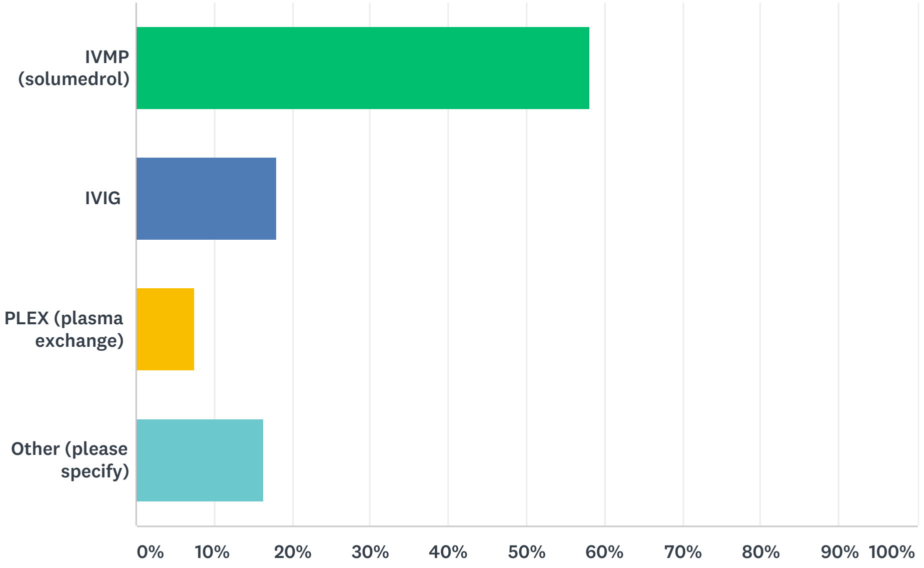

ANSWER CHOICES	RESPONSES	
IVMP (solumedrol)	58.21%	39
IVIG	17.91%	12
PLEX (plasma exchange)	7.46%	5
Other (please specify)	16.42%	11
TOTAL		67

Q9 For acute treatment of AE, which first line therapy would you choose in a patient with known or highly suspected paraneoplastic AE?

Answered: 68 Skipped: 0

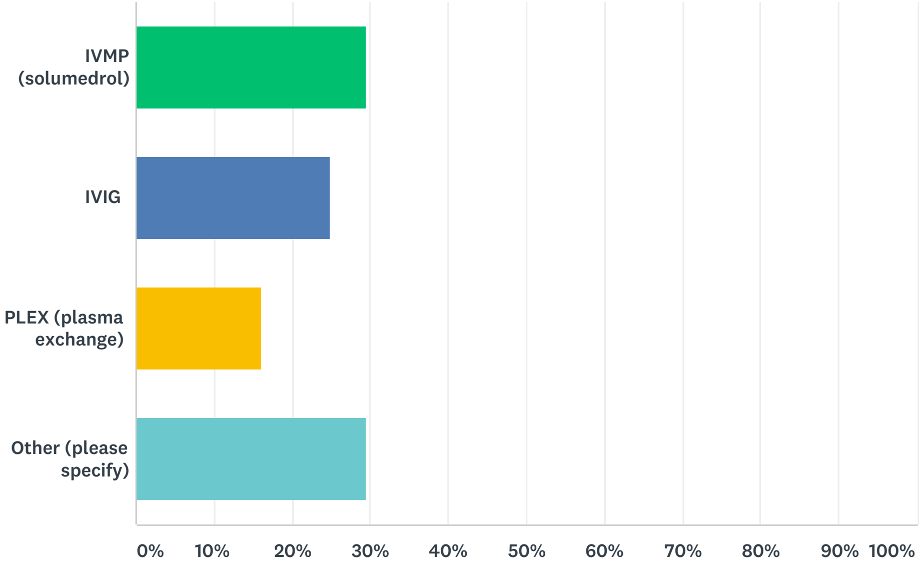

ANSWER CHOICES	RESPONSES	
IVMP (solumedrol)	29.41%	20
IVIG	25.00%	17
PLEX (plasma exchange)	16.18%	11
Other (please specify)	29.41%	20
TOTAL		68

Abboud H, *et al. J Neurol Neurosurg Psychiatry* 2021; 92:897–907. doi: 10.1136/jnnp-2020-325302

Q10 For acute treatment of AE, which first line therapy would you choose in a patient presenting solely with cognitive/psychiatric presentation?

Answered: 67 Skipped: 1

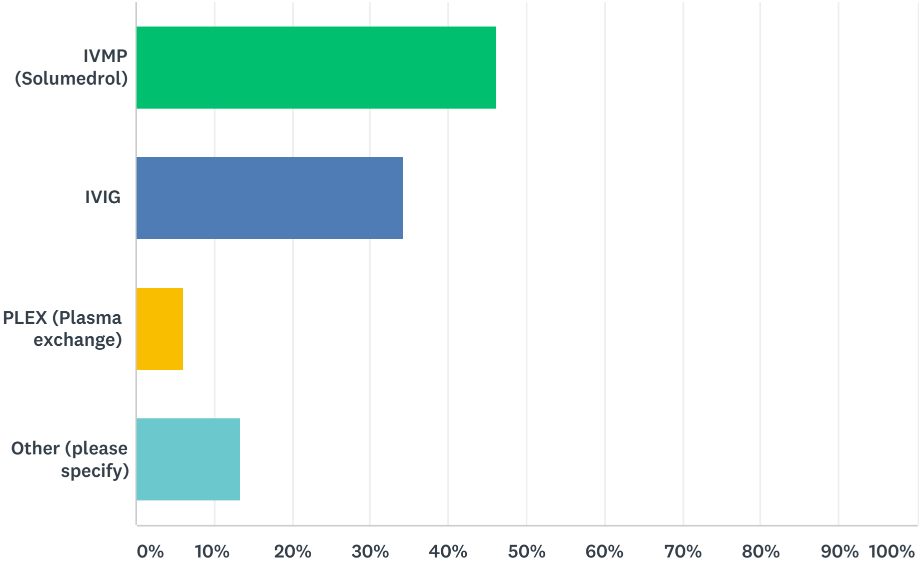

ANSWER CHOICES	RESPONSES	
IVMP (Solumedrol)	46.27%	31
IVIG	34.33%	23
PLEX (Plasma exchange)	5.97%	4
Other (please specify)	13.43%	9
TOTAL		67

Abboud H, *et al. J Neurol Neurosurg Psychiatry* 2021; 92:897–907. doi: 10.1136/jnnp-2020-325302

Q11 If no response to your initial first line therapy, what would you do next?

Answered: 68 Skipped: 0

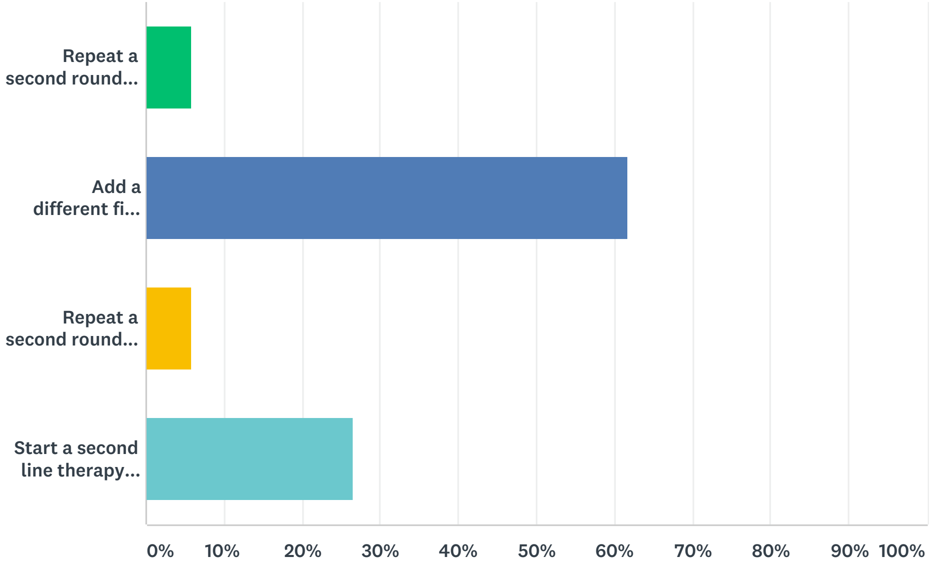

ANSWER CHOICES	RESPONSES	
Repeat a second round of the same first line therapy.	5.88%	4
Add a different first line therapy	61.76%	42
Repeat a second round of the same first line therapy and add a different first line therapy.	5.88%	4
Start a second line therapy directly.	26.47%	18
TOTAL		68

Q12 What second line therapy would you use in the acute setting if the patient is resistant to first line therapy?

Answered: 68 Skipped: 0

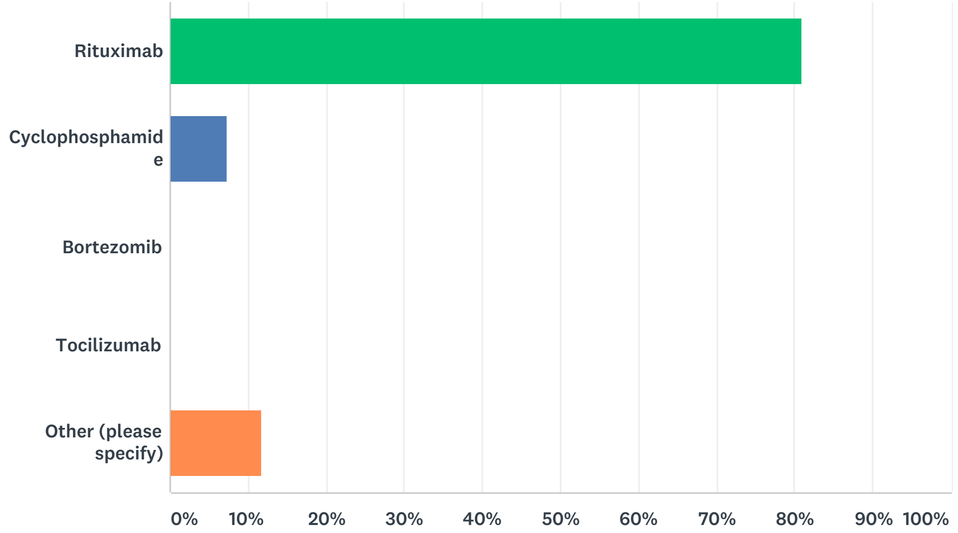

ANSWER CHOICES	RESPONSES	
Rituximab	80.88%	55
Cyclophosphamide	7.35%	5
Bortezomib	0.00%	0
Tocilizumab	0.00%	0
Other (please specify)	11.76%	8
TOTAL		68

Q13 How frequent do you use rituximab in the acute setting as a second line therapy?

Answered: 68 Skipped: 0

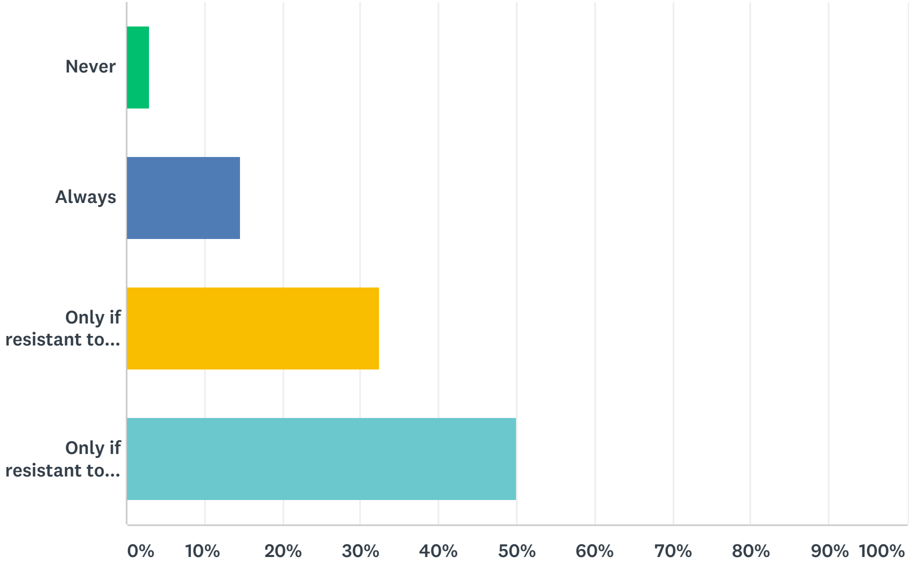

ANSWER CHOICES	RESPONSES	
Never	2.94%	2
Always	14.71%	10
Only if resistant to one modality of first line therapy.	32.35%	22
Only if resistant to combined modalities of first line therapies.	50.00%	34
TOTAL		68

Q14 What do you use as a bridging therapy in AE patients after acute treatment?

Answered: 68 Skipped: 0

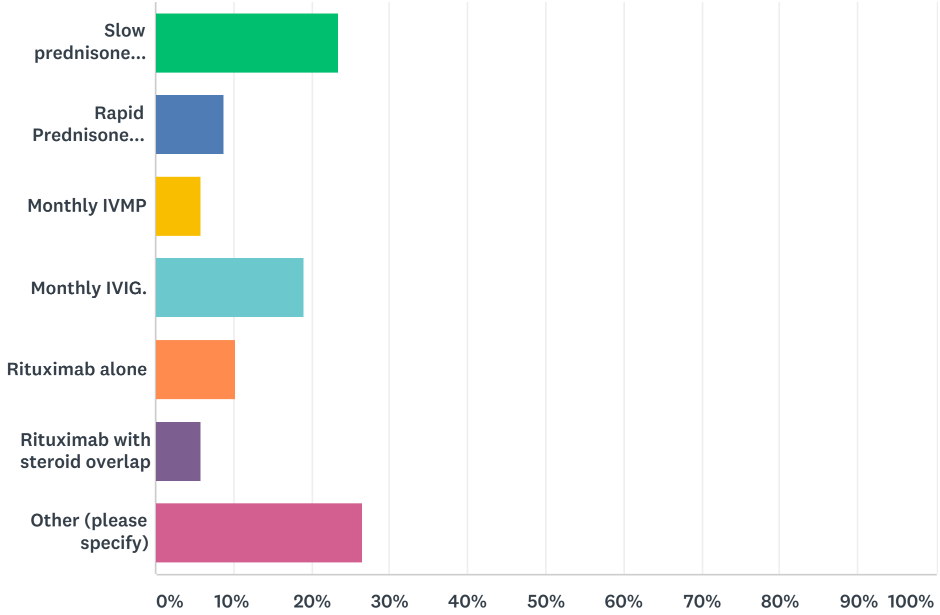

ANSWER CHOICES	RESPONSES	
Slow prednisone taper (months)	23.53%	16
Rapid Prednisone taper (weeks)	8.82%	6
Monthly IVMP	5.88%	4
Monthly IVIG.	19.12%	13
Rituximab alone	10.29%	7
Rituximab with steroid overlap	5.88%	4
Other (please specify)	26.47%	18
TOTAL		68

Q15 Which of the following clinical scenarios would you choose for long term or maintenance immunosuppression (check all that apply)?

Answered: 67 Skipped: 1

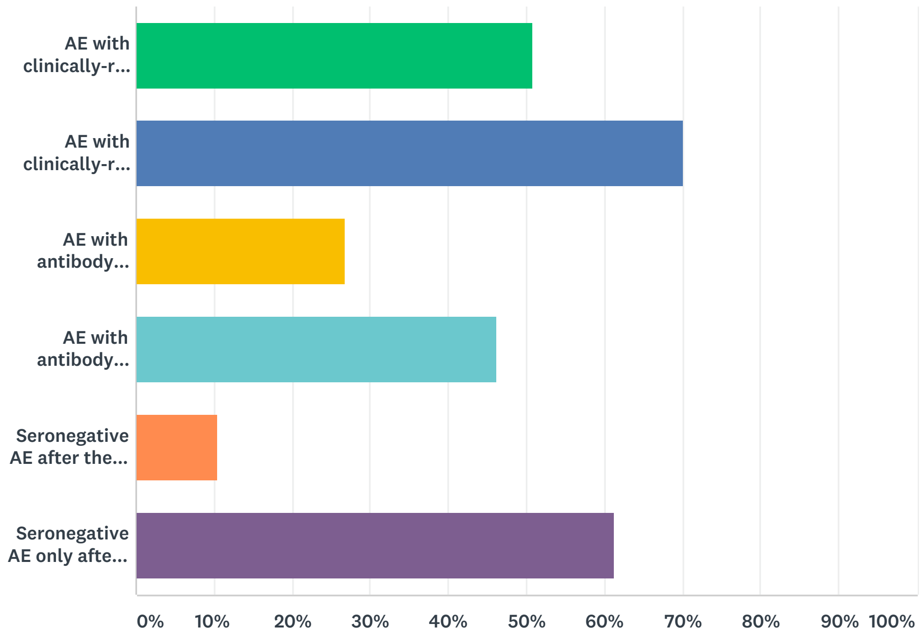

ANSWER CHOICES	RESPONSES	
AE with clinically-relevant Neuronal surface antibody after the first attack.	50.75%	34
AE with clinically-relevant Neuronal surface antibody only after a definitive second relapse	70.15%	47
AE with antibody against intracellular antigen after tumor treatment	26.87%	18
AE with antibody against intracellular antigen but negative tumor screen.	46.27%	31
Seronegative AE after the first attack.	10.45%	7
Seronegative AE only after a definitive second relapse.	61.19%	41
Total Respondents: 67		

Q16 How long would you keep a patient with recurrent AE on long term immunosuppression?

Answered: 68 Skipped: 0

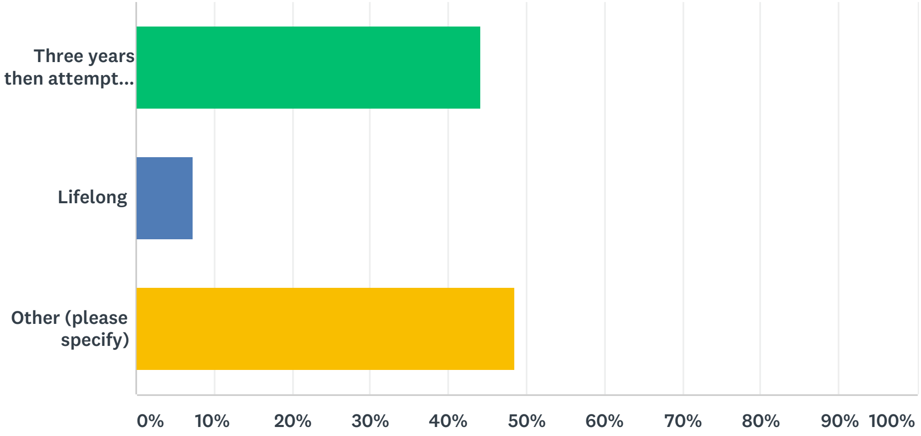

ANSWER CHOICES	RESPONSES	
Three years then attempt careful withdrawal of immunosuppression	44.12%	30
Lifelong	7.35%	5
Other (please specify)	48.53%	33
TOTAL		68

Q17 What agent would you choose for long term immunosuppression in recurrent AE?

Answered: 68 Skipped: 0

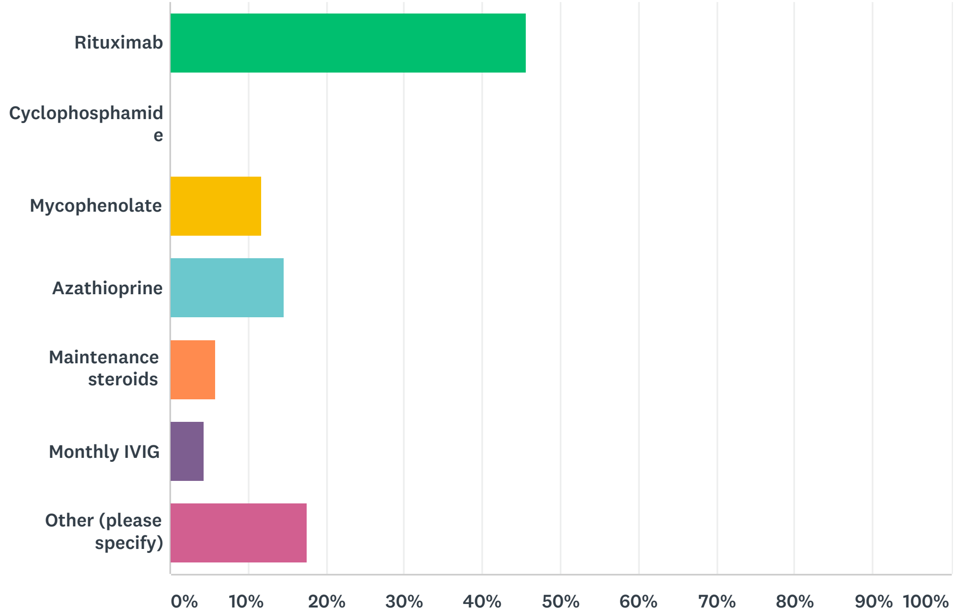

ANSWER CHOICES	RESPONSES	
Rituximab	45.59%	31
Cyclophosphamide	0.00%	0
Mycophenolate	11.76%	8
Azathioprine	14.71%	10
Maintenance steroids	5.88%	4
Monthly IVIG	4.41%	3
Other (please specify)	17.65%	12
TOTAL		68

Q18 After the initial screening, the European Federation of Neurological Societies recommend periodic cancer screening every 6-12 months for up to 4 years in patients with classical paraneoplastic syndromes, how frequent and for how long would you perform cancer screening in patients with AE associated with antibodies against neuronal surface antigens?

Answered: 67 Skipped: 1

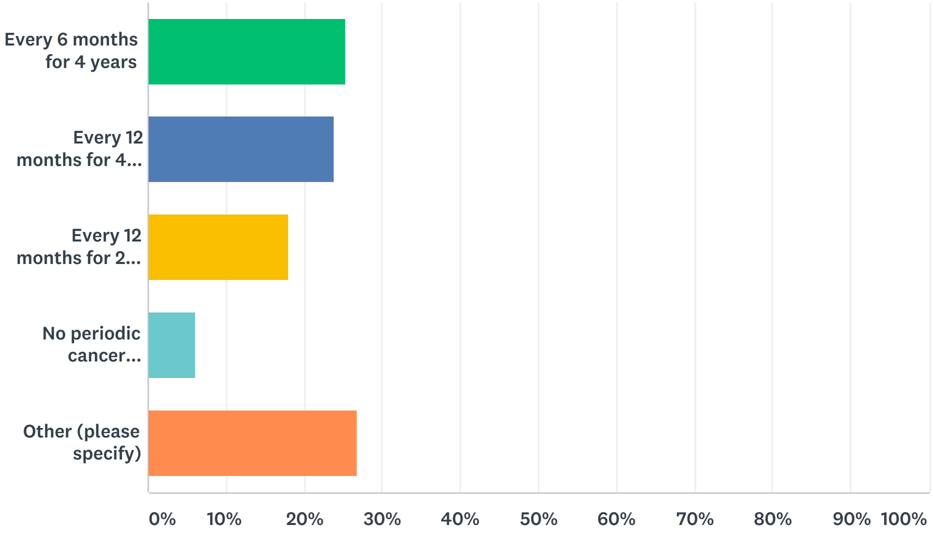

ANSWER CHOICES	RESPONSES	
Every 6 months for 4 years	25.37%	17
Every 12 months for 4 years	23.88%	16
Every 12 months for 2 years	17.91%	12
No periodic cancer screening necessary.	5.97%	4
Other (please specify)	26.87%	18
TOTAL		67

Q19 After the initial cancer screening, how frequent and for how long would you perform periodic cancer screening in patients with post-herpetic AE?

Answered: 67   Skipped: 1

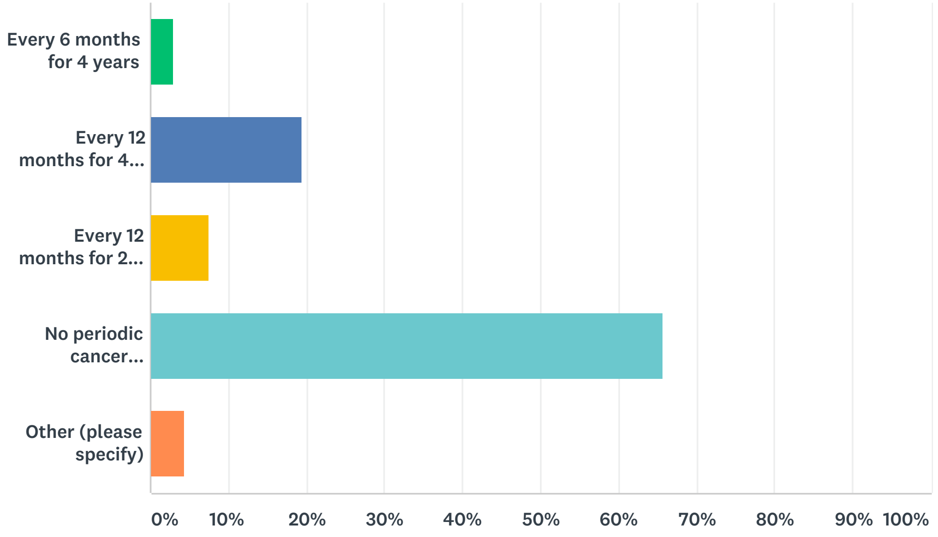

ANSWER CHOICES	RESPONSES	
Every 6 months for 4 years	2.99%	2
Every 12 months for 4 years	19.40%	13
Every 12 months for 2 years	7.46%	5
No periodic cancer screening necessary.	65.67%	44
Other (please specify)	4.48%	3
TOTAL		67

BMJ Publishing Group Limited (BMJ) disclaims all liability and responsibility arising from any reliance placed on this supplemental material which has been supplied by the author(s)

Q20 After the initial cancer screening, how frequent and for how long would you perform periodic cancer screening in patients with iatrogenic AE (in the setting of immune-modulating therapy other than checkpoint inhibitors)?

Answered: 66 Skipped: 2

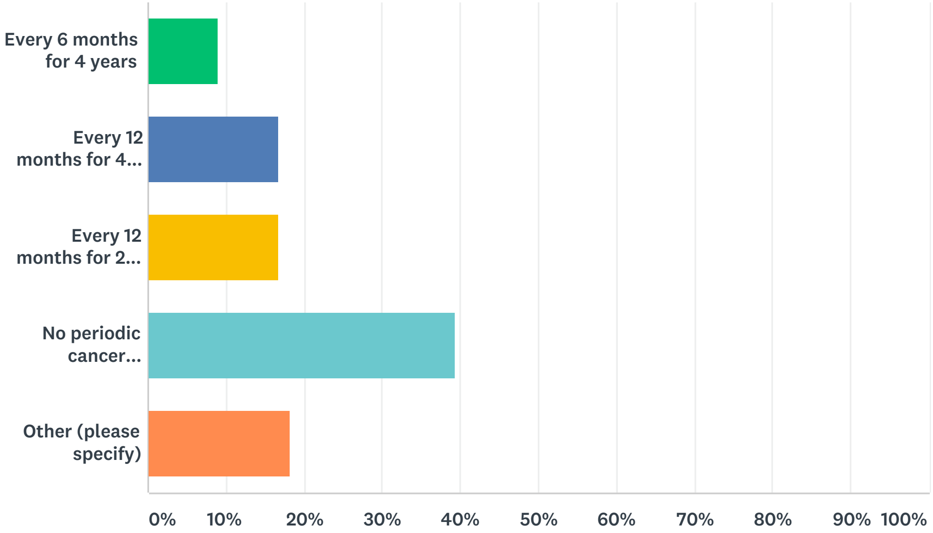

ANSWER CHOICES	RESPONSES	
Every 6 months for 4 years	9.09%	6
Every 12 months for 4 years	16.67%	11
Every 12 months for 2 years	16.67%	11
No periodic cancer screening necessary.	39.39%	26
Other (please specify)	18.18%	12
TOTAL		66

Abboud H, *et al. J Neurol Neurosurg Psychiatry* 2021; 92:897–907. doi: 10.1136/jnnp-2020-325302

Q21 After the initial cancer screening, how frequent and for how long would you perform periodic cancer screening in patients with seronegative limbic encephalitis?

Answered: 66 Skipped: 2

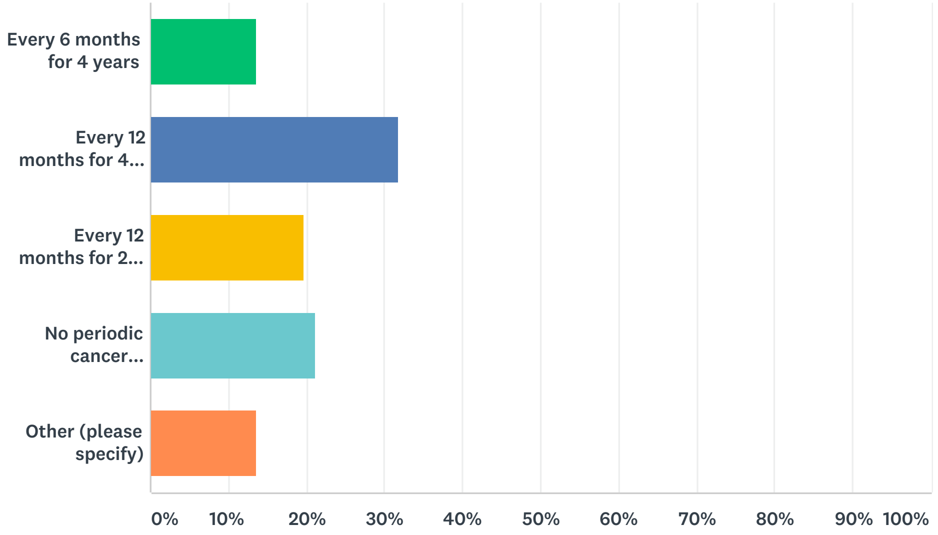

ANSWER CHOICES	RESPONSES	
Every 6 months for 4 years	13.64%	9
Every 12 months for 4 years	31.82%	21
Every 12 months for 2 years	19.70%	13
No periodic cancer screening necessary.	21.21%	14
Other (please specify)	13.64%	9
TOTAL		66

Q22 After the initial cancer screening, how frequent and for how long would you perform cancer screening in patients with seronegative AE other than limbic encephalitis (e.g. cortical, striatal, brainstem, cerebellar)?

Answered: 66 Skipped: 2

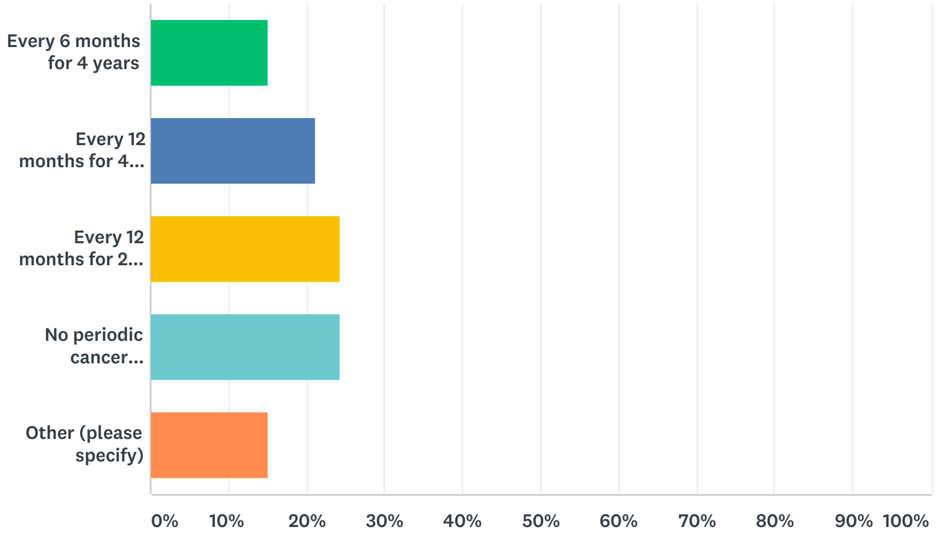

ANSWER CHOICES	RESPONSES	
Every 6 months for 4 years	15.15%	10
Every 12 months for 4 years	21.21%	14
Every 12 months for 2 years	24.24%	16
No periodic cancer screening necessary.	24.24%	16
Other (please specify)	15.15%	10
TOTAL		66

Q23 Do you order/review GRE or SWI sequences when evaluating brain MRI in a suspected case of AE?

Answered: 68 Skipped: 0

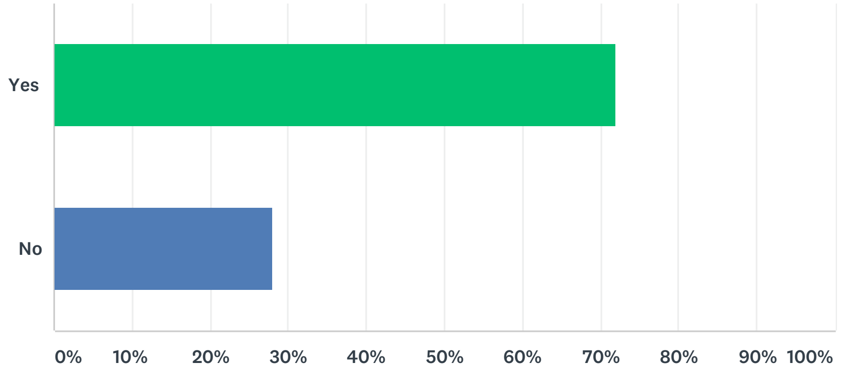

ANSWER CHOICES	RESPONSES	
Yes	72.06%	49
No	27.94%	19
TOTAL		68

Q24 If GRE or SWI sequences show hemorrhagic areas in the temporal lobes, what would be your provisional diagnosis?

Answered: 66 Skipped: 2

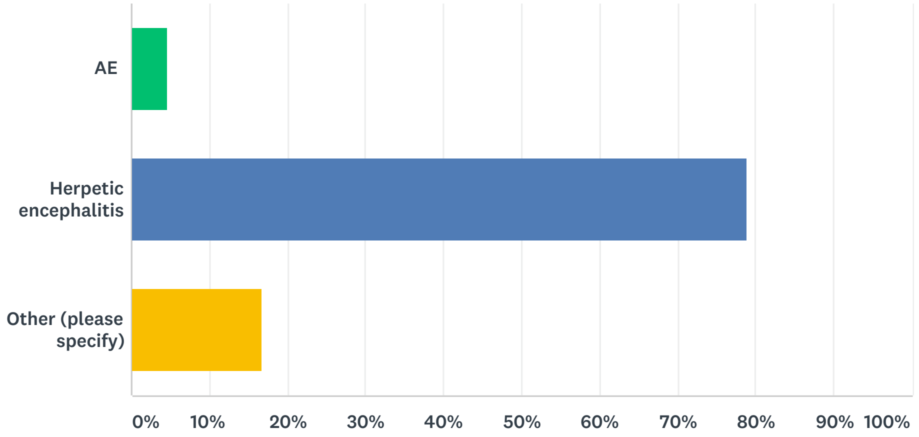

ANSWER CHOICES	RESPONSES	
AE	4.55%	3
Herpetic encephalitis	78.79%	52
Other (please specify)	16.67%	11
TOTAL		66

Abboud H, *et al. J Neurol Neurosurg Psychiatry* 2021; 92:897–907. doi: 10.1136/jnnp-2020-325302
