# Supplementary material for: Autoimmune encephalitis: proposed recommendations for symptomatic and long-term management
Source: J Neurol Neurosurg Psychiatry. 2021 Mar 1;92(8):897–907. doi: 10.1136/jnnp-2020-325302 (PMC8292591; doi:10.1136/jnnp-2020-325302)
Supplement: Supplementary data [file jnnp-2020-325302supp003.pdf]

Supplemental box1: Diagnostic criteria for definite autoimmune limbic encephalitis

Diagnosis can be made when all four* of the following criteria have been met:

1 Subacute onset (rapid progression of less than 3 months) of working memory deficits, seizures, or psychiatric symptoms suggesting involvement of the limbic system

2 Bilateral brain abnormalities on T2-weighted fluid-attenuated inversion recovery MRI highly restricted to the medial temporal lobes†

3 At least one of the following:

• CSF pleocytosis (white blood cell count of more than five cells per mm3)

• EEG with epileptic or slow-wave activity involving the temporal lobes

4 Reasonable exclusion of alternative causes

*If one of the first three criteria is not met, a diagnosis of defi nite limbic encephalitis can be made only with the detection of antibodies against cell-surface, synaptic, or onconeural proteins.

†1^{8}Fluorodeoxyglucose (1^{8}F-FDG) PET can be used to fulfil this criterion. Results from studies from the past 5 years suggest that 1^{8}F-FDG-PET imaging might be more sensitive than MRI to show an increase in FDG uptake in normal-appearing medial temporal lobes.

Adapted with permission from Graus F, Titulaer MJ, Balu R, et al. A clinical approach to diagnosis of autoimmune encephalitis. Lancet Neurol. 2016;15(4):391-404. doi:10.1016/S1474-4422(15)00401-9
