# Supplementary material for: Autoimmune encephalitis: proposed recommendations for symptomatic and long-term management
Source: J Neurol Neurosurg Psychiatry. 2021 Mar 1;92(8):897–907. doi: 10.1136/jnnp-2020-325302 (PMC8292591; doi:10.1136/jnnp-2020-325302)
Supplement: Supplementary data [file jnnp-2020-325302supp004.pdf]

Supplemental box2: Criteria for autoantibody-negative but probable autoimmune encephalitis

Diagnosis can be made when all four of the following criteria have been met:

1 Rapid progression (less than 3 months) of working memory deficits (short-term memory loss), altered mental status, or psychiatric symptoms

2 Exclusion of well-defined syndromes of autoimmune encephalitis (e.g., typical limbic encephalitis, Bickerstaff ʹs brainstem encephalitis, acute disseminated encephalomyelitis)

3 Absence of well characterized autoantibodies in serum and CSF, and at least two of the following criteria:

• MRI abnormalities suggestive of autoimmune encephalitis*

• CSF pleocytosis, CSF-specific oligoclonal bands or elevated CSF IgG index, or both*

• Brain biopsy showing inflammatory infiltrates and excluding other disorders (e.g., tumor)

4 Reasonable exclusion of alternative causes

*Some inherited mitochondrial and metabolic disorders can present with symmetric or asymmetric MRI abnormalities

Adapted with permission from Graus F, Titulaer MJ, Balu R, et al. A clinical approach to diagnosis of autoimmune encephalitis. Lancet Neurol. 2016;15(4):391-404. doi:10.1016/S1474-4422(15)00401-9
